# Excited-state proton-coupled electron transfer within ion pairs†

**DOI:** 10.1039/c9sc04941j

**Published:** 2020-03-03

**Authors:** Wesley B. Swords, Gerald J. Meyer, Leif Hammarström

**Affiliations:** Department of Chemistry, Ångström Laboratories, Uppsala University Box 523 SE75120 Uppsala Sweden Leif.Hammarstrom@kemi.uu.se; Department of Chemistry, University of North Carolina at Chapel Hill Chapel Hill 27599 USA

## Abstract

The use of light to drive proton-coupled electron transfer (PCET) reactions has received growing interest, with recent focus on the direct use of excited states in PCET reactions (ES-PCET). Electrostatic ion pairs provide a scaffold to reduce reaction orders and have facilitated many discoveries in electron-transfer chemistry. Their use, however, has not translated to PCET. Herein, we show that ion pairs, formed solely through electrostatic interactions, provide a general, facile means to study an ES-PCET mechanism. These ion pairs formed readily between salicylate anions and tetracationic ruthenium complexes in acetonitrile solution. Upon light excitation, quenching of the ruthenium excited state occurred through ES-PCET oxidation of salicylate within the ion pair. Transient absorption spectroscopy identified the reduced ruthenium complex and oxidized salicylate radical as the primary photoproducts of this reaction. The reduced reaction order due to ion pairing allowed the first-order PCET rate constants to be directly measured through nanosecond photoluminescence spectroscopy. These PCET rate constants saturated at larger driving forces consistent with approaching the Marcus barrierless region. Surprisingly, a proton-transfer tautomer of salicylate, with the proton localized on the carboxylate functional group, was present in acetonitrile. A pre-equilibrium model based on this tautomerization provided non-adiabatic electron-transfer rate constants that were well described by Marcus theory. Electrostatic ion pairs were critical to our ability to investigate this PCET mechanism without the need to covalently link the donor and acceptor or introduce specific hydrogen bonding sites that could compete in alternate PCET pathways.

## Abstract 2

Electrostatic ion pairs provide a general method to study excited-state proton-coupled electron transfer. A PT_a_ET_b_ mechanism is identified for the ES-PCET oxidation of salicylate within photoexcited cationic ruthenium–salicylate ion pairs.

## Graphical abstract

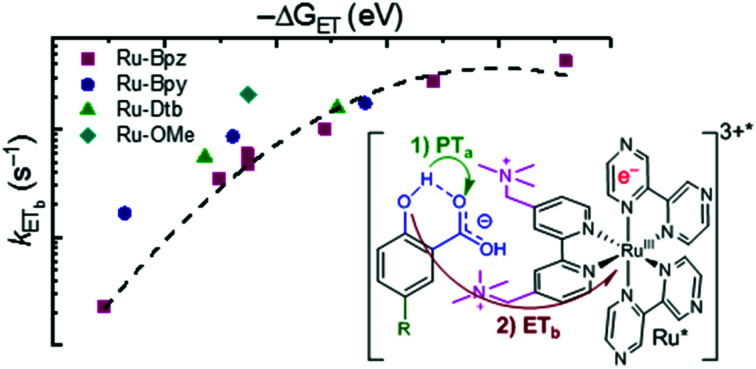

## Introduction

The creation of energy-rich fuels from small molecules is dependent upon the ability to effectively couple proton and electron transfer. There is growing interest in the use of solar energy to drive these proton-coupled electron transfer (PCET) reactions.^1–4^ To accomplish this feat, molecular systems that effectively couple light energy to proton and electron transfer are needed. Two approaches that have been utilized to couple light to PCET include the flash-quench technique, where photoexcitation of a sensitizer is followed by rapid electron-transfer quenching by a redox mediator to yield an oxidized or reduced sensitizer. This oxidized or reduced sensitizer then initiates a thermal PCET reaction with a secondary substrate.^5^ Alternatively, the excited sensitizer can directly participate in the PCET reaction.^6^ While excited-state PCET (ES-PCET) has been less explored than thermal PCET, there are a growing number of fundamental and application-based studies in the field.^1,6–9^

**Scheme 1:**
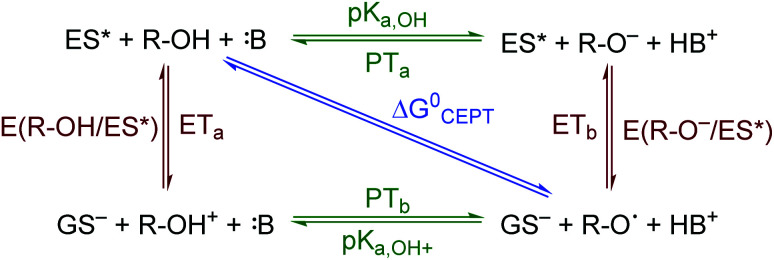
ES-PCET reaction diagram. ES* is the excited sensitizer, GS^−^ is the reduced sensitizer, R-OH is the protonated substrate, and B is a base.

ES-PCET reactions occur through three distinct mechanisms, Scheme 1,^1,10^ (1) stepwise electron transfer-proton transfer (ET_a_PT_b_), (2) stepwise proton transfer-electron transfer (PT_a_ET_b_), and (3) concerted electron-proton transfer (CEPT), in which the proton and electron are transferred in the same step with a common transition state. The concerted mechanism is expected to be more valuable for selective catalysis as it avoids high energy intermediates^11^ and may occur with lower reaction barriers.^12^ However, the kinetic penalty associated with proton tunnelling and the need to bring three reagents together may favor the stepwise pathways.^13–15^

In this work, we show electrostatic interactions provide a general means to investigate ES-PCET reactions without the limitations imposed by covalent or hydrogen bonds. Upon excitation of ion pairs formed between cationic ruthenium compounds and anionic salicylate derivatives, quenching of the ruthenium excited state proceeded through a PCET mechanism. One-electron oxidation of the ion-paired salicylate by the photoexcited ruthenium was coupled to proton transfer within an internal salicylate hydrogen bond. Thus, upon light excitation, the electron and proton transfer occurs entirely within the ion pair, removing any need for reactant diffusion.

Salicylate was chosen as the counteranion in this study because it contains an asymmetric internal hydrogen bond between a phenolic-OH and a carboxylate functional group.^16–18^ It has been well documented under aqueous conditions that, upon one-electron oxidation, transfer of the localized phenolic-OH proton to the carboxylate forms a carboxylic acid and phenoxyl radical. In flash-quench studies, PCET proceeded through a CEPT mechanism, where intramolecular proton transfer occurred in concert with electron transfer to an oxidized ruthenium(iii) center.^19,20^ With the need for more non-polar, aprotic organic solvents like acetonitrile or dichloromethane to assist the preorganization of ion-pairs,^21,22^ we hoped the use of salicylate would bias our system towards a concerted mechanism while providing a scaffold to systematically study salicylate oxidation under non-aqueous conditions.

The use of undirected ion pairs presents a new approach to the field of ES-PCET. Almost all studies to date of ES-PCET fall under three categories. (1) Diffusional pathways, where the electron and proton transfer components react through a diffusional interaction.^23–25^ (2) Covalent bonds, where the sensitizer and PCET reactant are bound through an organic framework.^9,26–30^ (3) Hydrogen bonds, where the sensitizer and PCET reactant are brought together through a hydrogen bond.^3,31–34^ The use of covalent and hydrogen bonds in ES-PCET has facilitated large gains in the fundamental understanding of ES-PCET. One large convenience afforded through the latter two methods is that the removal of reactant diffusion allows direct measurement of the ES-PCET reaction rate constants. However, these methods also have limitations. The covalent systems are often synthetically difficult to prepare. The hydrogen-bond systems inherently require specific functionality to form the hydrogen bond interface, and the association constant is small (*K* ≤ 10^3^ M^−1^) in most cases. Salt-bridged systems have achieved association constants on the order of *K* ≈ 10^2^ to 10^3^ M^−1^ in high dielectric solvents (DMSO) and *K* ≈ 10^4^ to 10^5^ M^−1^ in lower polarity solvents (THF, CH_2_Cl_2_), but the studies focused on variations of the salt bridge and not on systematic variations of the donor–acceptor components themselves.^31,33–37^ Thus, there have been no systematic ES-PCET studies of donor–acceptor systems bound by general, non-directional ion pairing. Through removal of both the synthetic difficulty of covalent systems and required hydrogen bond functionality, electrostatic ion pairs may provide a more general methodology toward fundamental and application-oriented ES-PCET studies.

To our knowledge only one study has potentially measured ES-PCET in ion-pairs without an *inter*molecular hydrogen bond. Auodia and Rodgers reported that electron-transfer rate constants within ion pairs of a tyrosine terminated anionic polypeptide chain and a tetracationic porphyrin sensitizer varied with the pH of the aqueous solution.^38,39^ While they assigned these pH dependant rate constants to electron transfer, a re-analysis of the data supports that the reaction most likely occurred through an ES-PCET mechanism with the solution buffer (see ESI†).

Herein, ion pairing is used to study a fundamental ES-PCET reaction between cationic ruthenium sensitizers and anionic salicylate derivatives. Pre-association of the ion-paired complex occurred in acetonitrile solution with equilibrium constants on the order of 10^5^. Following photoexcitation of the ion-pairs, unimolecular quenching of the ruthenium excited state corresponded to *intra*-ion-pair PCET. Importantly, as with prior covalent and hydrogen-bond systems, these rate constants could be measured without complications from diffusion. Systematic variation of the driving force for PCET allowed analysis of the ES-PCET mechanism, which was ascribed to a sequential PT_a_ET_b_ reaction. A pre-equilibrium model of this mechanism provided a rare example of rate constants near the Marcus barrierless region in a PCET reaction, *i.e.* a region of driving force where the rate changes only weakly, or not at all. At even higher driving force, the rate decreases with increasing driving force, in the so-called Marcus inverted region.^40^ This behaviour was clearly demonstrated more than 30 years ago for ground-state electron transfer (charge shift) by Closs, Miller, and co-workers,^41^ and for photochemical charge recombination by Wasielewski and co-workers.^42^ For PCET reactions, however, inverted region behaviour was shown only recently,^9,30^ and even near-barrierless PCET reactions are rare.^43^ Ion pairing was critical to our ability to investigate this mechanism by reducing the reaction order for PCET and provides a broad, general methodology that will be of interest in future application and mechanistic PCET investigations.

## Experimental

### Materials

Acetonitrile (spectroscopic grade, Alfa Aesar) was used as received. All seven salicylate derivatives (>97%) were purchased from Sigma Aldrich and used as received. The ruthenium compounds utilized were all synthesized for prior studies.^44–46^ Tetrabutylammonium 30-hydrate (Sigma Aldrich, >98%) was used as received.

### Electrochemistry

Square-wave and cyclic voltammetery were collected on a autolab potentiostat in a standard 3-electrode set-up. A platinum disk was used as the working electrode, a platinum rod as the counter electrode, and a Ag/AgNO_3_ electrode was used as a pseudo reference electrode. An inert electrolyte composed of 100 mM TBAClO_4_/CH_3_CN was used. All potentials were externally referenced to Fc/Fc^+^ (630 mV *vs.* NHE).^47^

### UV-visible spectroscopy

UV-vis absorption spectra were acquired on a Varian Cary 50 UV-vis spectrophotometer in 1 cm path length spectrophotometric quartz cuvettes. Resolution of 1 nm was used.

### Time-resolved photoluminescence and nanosecond transient absorption spectroscopy

Time-resolved photoluminescence and nanosecond transient absorption (TA) single wavelength kinetic data were collected on an Applied Photophysics spectrometer. Optical excitation was afforded by an OPO (opotek) pumped by a Q-switched, frequency tripled (355 nm) Nd:YAG laser (Quantel, Brilliant B). Pulses had an ∼7 ns FWHM at 460 nm (*c.a.* 10 mJ per pulse). A pulsed Xenon lamp of an Applied Photophysics LKS60 setup provided probe light that was passed through 1 cm^2^ quartz cuvette 90° to the laser and through a monochromator before hitting the P928 photomultiplier. For photoluminescence measurements the probe lamp was not used, and photoluminescence was detected at 90° to the incident laser through the same detection system. The PMT signal was converted and digitized using an HP Infinitum S5 digital oscilloscope (2G samples per s). Transient absorption traces were generated from the raw data using the LKS60 software.

Full transient absorption spectra were acquired on a Spectra Physics Quanta-Ray system with a frequency doubled (532 nm), Q-switch Nd:YAG laser. The pulse laser was connected to a transient absorption detection system (Edinburgh Instruments), equipped with a monochromator and a pulsed Xe arc lamp. A transient absorption spectrum of the sample was collected at 90° to the incident laser by a Tektronix 500 MHz digital oscilloscope coupled to a CCD camera. The output was processed with Edinburgh Instruments' L900 software. All data analysis was performed on OriginPro 2016 and 2017 software.

### Titrations

All samples were purged with nitrogen for 5–10 minutes prior to measurements and a flow of nitrogen was maintained over the samples during data collection. Ruthenium concentrations were held around 20–30 μM. Stock solutions of the salicylate derivatives were prepared at 5–10 mM and were titrated into the ruthenium samples in 10–100 μL amounts.

### Cage escape quantum yields

Cage escape quantum yields were determined from the nanosecond transient absorption experiments through eqn (1). Ru(bpy)_3_^2+^ was utilized as an actinometer assuming a unity yield of intersystem crossing. The Δ*ε*_450_ between the ground-state Ru(bpy)_3_^2+^ and the excited-state Ru(bpy)_3_^2+*^ was −1.5 × 10^4^ M ^−1^ cm^−1^,^48^ and Δ*ε*_510_ between the ground-state **Ru-Bpz4+** and the reduced **Ru-Bpz3+** was 1.05 × 10^4^ M^−1^ cm^−1^. Salicylate concentrations of ∼75 μM were utilized as at this concentration >98% of the photoluminescence had been quenched.1
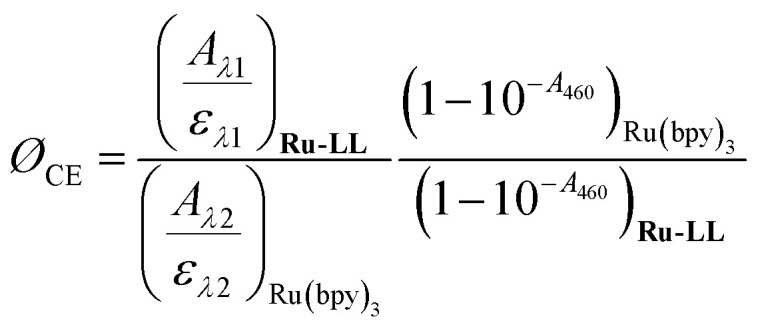


## Results and discussion

### Characterization of salicylate anions

**Scheme 2:**
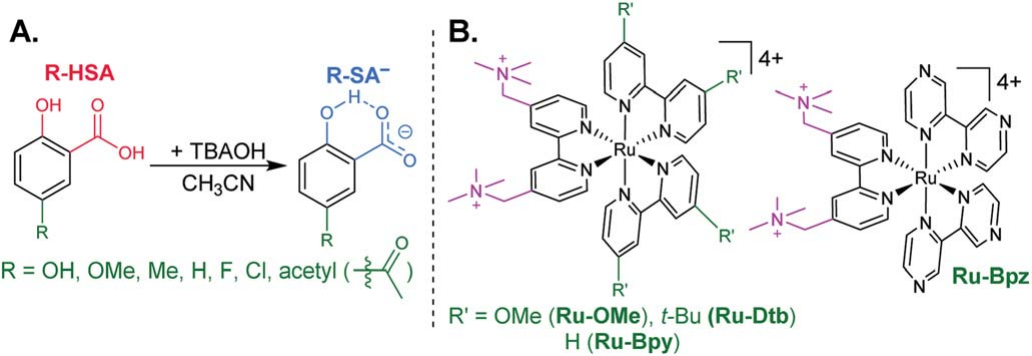
(A) Salicylate derivatives and (B) tetracationic ruthenium sensitizers.

**Fig. 1:**
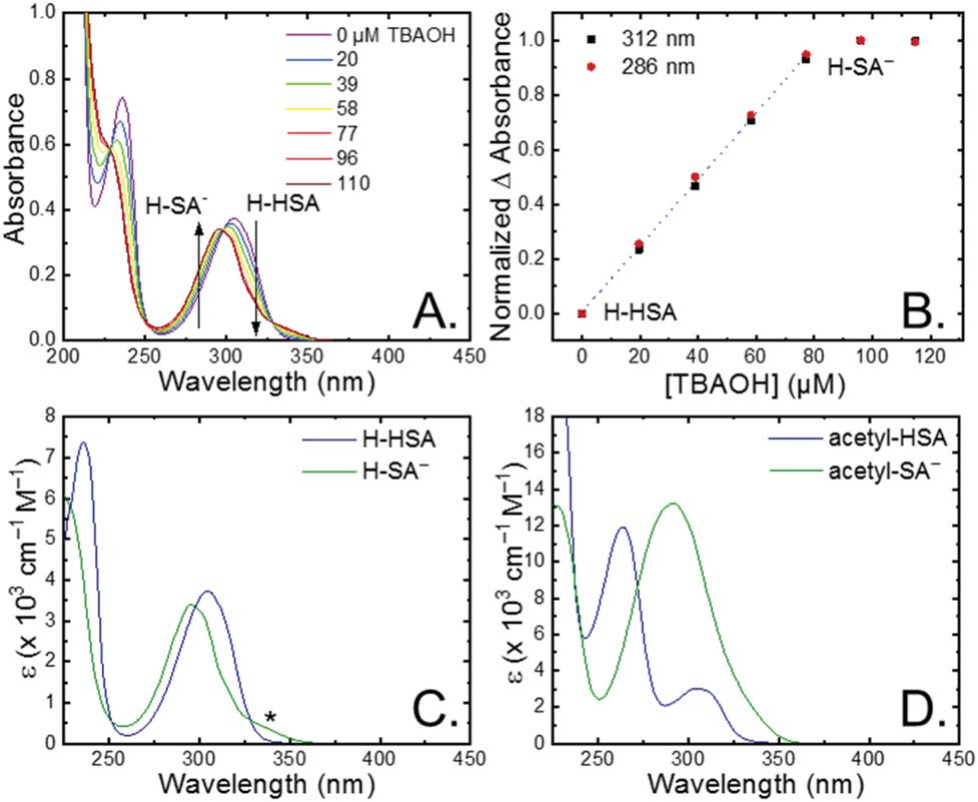
(A) UV-vis absorption spectral changes upon the addition of tetrabutylammonium hydroxide to an ∼80 μM solution of **H-HSA** in CH_3_CN. (B) Normalized change in absorbance monitored at the absorbance maxima for **H-HSA** and **H-SA−**. Dotted line is a linear fit through the first five data points. (C and D) Extinction coefficient spectrum for **R-HSA** and **R-SA−** when (C) R = H or (D) R = acetyl. Asterisk marks the tautomer absorbance.

The seven salicylic acids (**R-HSA**), where *R* is the functional group *para*- to the phenol (**OH-**, **OMe-**, **Me-**, **H-**, **F-**, **Cl-**, **acetyl-**), were readily deprotonated in acetonitrile (CH_3_CN) through the *in situ* addition of tetrabutylammonium hydroxide 30-hydrate (TBAOH) to form the salicylate anions (**R-SA−**), Scheme 2. The deprotonation was monitored by UV-vis spectroscopy. Hypsochromic shifts of 10–20 nm (∼0.1–0.18 eV) were accompanied by a slight decrease in the absorption intensity. This change in absorption was linear with respect to the TBAOH concentration up to one equivalent, upon which the spectral changes saturated. A set of isosbestic points was maintained throughout the titration indicative of clean conversion to the deprotonated anion. Fig. 1A–C shows the deprotonation for **H-HSA** to **H-SA−**, along with the extinction coefficient spectra of each entity. **Acetyl-HSA** showed different spectroscopic changes than the other six **R-HSA** derivatives. Upon the addition of TBAOH, a significant increase in the low-energy absorption intensity occurred, Fig. 1D.

The p*K*_a_ associated with this deprotonation in CH_3_CN was determined through the spectrophotometric titration of 2,4-bis(tetramethylphenyl)-7-(dimethylamino)quinoline (p*K*_a_ = 15.2 in CH_3_CN).^49^ The measured p*K*_a_ of **H-HSA** (16.7) aligned well with that previously reported as 16.7.^50^ For **OH-**, **OMe-**, and **Me-HSA**, the p*K*_a_s were found in the range of 16.9–16.6. Whereas **F-**, **Cl-**, and **acetyl-HSA** were more acidic, in the range 15.8–15.4. The p*K*_a_ values are presented in Table 1.

**Table 1:** Redox potentials, photophysical properties, p*K*_a_s, and tautomer equilibrium constants for **R-SA−**

R	*E*_app_(**R-SAOH−**/**R-SA˙O**) (V *vs.* NHE)	*λ*_max,_ **R-HSA** (nm), *ε***R-HSA** (M^−1^ s^−1^)	*λ*_max,_ **R-SA−** (nm), *ε***R-SA−** (M^−1^ s^−1^)	p*K*_a_	*K*_EQ,Taut_
OH	0.56^a^	335, 4200	320, 4100	16.9	0.03
OMe	0.79	333, 4200	318, 4100	16.6	0.04
Me	0.97	314, 3800	304, 3600	16.9	0.07
H	1.10	304, 3700	295, 3400	16.7^b^	0.10
F	1.11	314, 4400	305, 4100	15.8	0.07
Cl	1.16	316, 3500	307, 3200	15.6	0.11
Acetyl	1.30	305, 3100	292, 13 200	15.4	1.2

a Oxidation of **OH-SA−** was quasi-reversible. The value reported is the *E*_1/2_.

b Aligns with literature value of 16.7.^50^

**Fig. 2:**
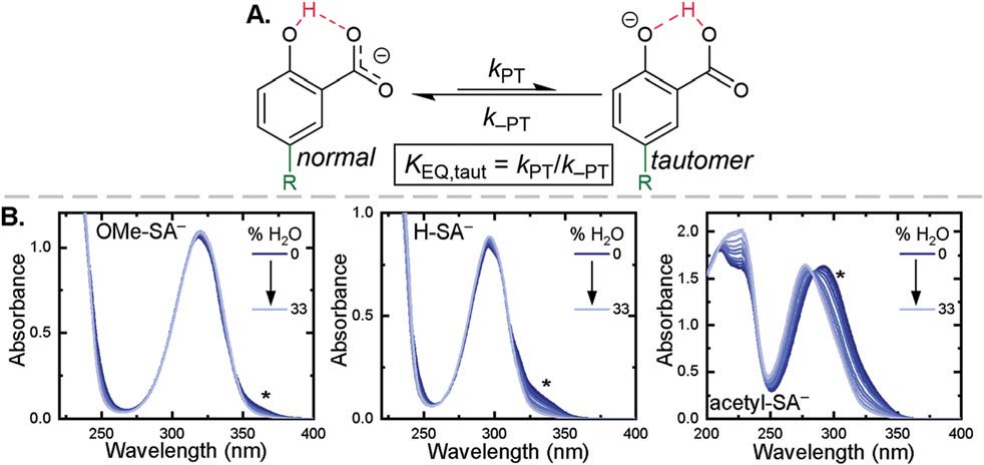
(A) Salicylate proton-transfer tautomer. The normal and tautomeric forms are labelled. (B) Absorption spectral changes upon the addition of increasing amounts of H_2_O to CH_3_CN solutions of the indicated salicylate derivatives, ∼250 μM. Asterisks mark the tautomer absorbance.

Upon deprotonation, a small red-absorbing shoulder appeared at ∼340 nm for all the **R-SA−** compounds, except **acetyl-SA−**. This absorption was previously identified for **H-SA−** in acetonitrile and ethanol as a proton-transfer tautomer, Fig. 2A.^16,17^ In this tautomeric form, the proton is localized on the carboxylate functional group instead of the phenolic oxygen. This tautomeric form was not expected at the outset of this study as previous PCET experiments in solely H_2_O did not observe this tautomer.^19^ However, in non-polar organic solvents this tautomer has been characterized in both *intra*- and *inter*molecular phenol-carboxylate systems.^16,17,51^ In the *inter*molecular studies, non-polar solvents were suggested to better stabilize larger, delocalized anions, such as expected for a phenolate, and the equilibrium between the normal and tautomeric form was found to shift towards the tautomer as solvent polarity decreased.^51^ As acetonitrile is a reasonably polar organic solvent, only a small amount of tautomer absorbance was found for the salicylate derivatives studied, barring **acetyl-SA−**.

The addition of water to a CH_3_CN solution of salicylate (**H-SA−**) was shown to shift the equilibrium towards the normal isomer (protonated phenol).^16^ Therefore, to show that the low intensity absorbance measured for the expanded series of **R-SA−** derivatives was due to this tautomer, titrations with deionized water were performed, Fig. 2. A loss of the tautomer absorbance was correlated to a growth of the normal isomer absorbance and confirmed our assignment of the low-energy absorbance to the tautomer.

The absorption spectra of the salicylate derivatives in CH_3_CN were fit with the sum of two Gaussian functions to approximate the absorbance of the tautomer. It was assumed that the extinction coefficient of the two species at the maximum absorbance was identical, as was previously done for similar phenol/carboxylate tautomers.^51^ Therefore, the ratio between the maxima absorbances gave the equilibrium constant for the tautomerization (*K*_EQ,Taut_), Table 1. A measurable increase in the tautomer equilibrium constant coincided with increased electron withdrawing character of the functional group *para*- to the phenolic-OH. This substitution *para*- to the phenolic-OH group is known to decrease the intrinsic p*K*_a_ of the phenolic-OH.^18^ This decreases the difference in p*K*_a_ between the carboxylate and phenolic-OH, thus lowering the driving force for proton transfer and increasing the concentration of tautomer in solution.

**Fig. 3:**
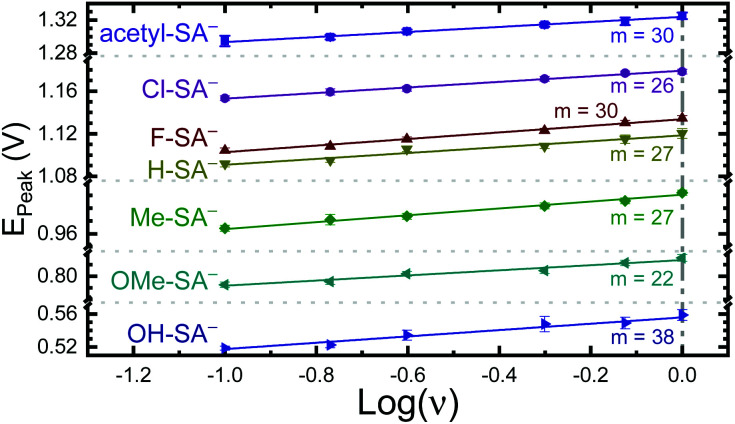
Peak potential from irreversible cyclic voltammograms of the indicated **R-SA−**s *vs.* the log of the scan rate (*ν*). The indicated slopes (*m*) are given in mV per decade. Note the peak potential axis is broken in multiple places for clarity. Error bars are included and in most cases are similar in size to the symbol.

The apparent reduction potentials of the salicylate derivatives were determined through cyclic voltammetry, *E*_app_(**R-SA˙O**/**R-SAOH−**) where **R-SAOH−** is the salicylate derivative before oxidation and **R-SA˙O** is the oxidized **R-SA−** that has undergone an intramolecular proton transfer to the carboxylate functional group. The oxidation of the **R-SA−** compounds was completely irreversible as expected for phenolic compounds that undergo an irreversible dimerization after oxidation.^52^ Also, because the proton transfer is coupled with electron transfer in the oxidation, a true one-electron reduction potential *E*°(**R-SAOH**/**R-SAOH−**) for **R-SA−** could not be measured. However, the apparent reduction potential for the PCET reaction was estimated through a scan rate (*ν*) dependence, *E*_app_(**R-SA˙O**/**R-SAOH−**).^52–54^ A plot of log(*ν*) *versus* the oxidative peak potential was linear with slopes of 20–30 mV per decade, within a reasonable deviation from the theoretical 19.7 mV per decade expected for a PCET reaction, Fig. 3.^52^ The *y*-intercept was corrected for scan rate independent variables and the apparent PCET reduction potentials (*E*_app_) are presented in Table 1.

### Ion pair formation

Typical ruthenium tris-bipyridyl complexes have a 2+ cationic charge associated with the *d*^6^ ruthenium center. The dicationic 4,4′-bis(trimethylaminomethyl)-2,2′-bipyridine (*tmam*) ligand has been used to increase the charge of the polypyridyl compounds by 2+ per ligand. This has been shown to enhance the formation of ion pairs between cationic ruthenium polypyridyl compounds and anions in acetonitrile (CH_3_CN).^23,44,55–57^ The four ruthenium compounds utilized in this study follow the common structure of [Ru(tmam)(LL)_2_](PF_6_)_4_, where LL was 4,4′-dimethoxy-2,2′-bipyridine (OMe), 4,4′-di-*tert*-butyl-2,2′-bipyridine (Dtb), 2,2′-bipyridine (Bpy), and 2,2′-bipyrazine (Bpz). Herein, the compounds will be denoted **Ru-LL** where LL is the short name of the derivatized bipyridine or bipyrazine ligand (Scheme 2B).

The electrochemical properties of the **Ru-LL** compounds, except **Ru-Bpz** which was previously reported,^58^ were determined through square-wave voltammetry in 0.1 M TBAClO_4_/CH_3_CN solution. The one-electron oxidation of **Ru-LL** (**Ru-LL5+/4+**) was formally a ruthenium centered oxidation, Ru^III/II^. These potentials were measured to be between 1.39 and 2.10 V, with a negative shift of the potential in the order **Ru-OMe** < **Ru-Dtb** < **Ru-Bpy** < **Ru-Bpz**. This trend followed the electron donating ability of the ancillary ligands. The first one-electron reduction of ruthenium polypyridyl compounds, **Ru-LL4+/3+** has been shown to occur at the ligand that is the best π-acceptor.^59,60^ For **Ru-Bpz** this was the Bpz ligand, whereas for **Ru-OMe**, **Ru-Dtb**, and **Ru-Bpy**, the reduction was localized to a tmam ligand. The reduction of **Ru-Bpz** was reversible and ∼300 mV more positive than the reduction of the other three compounds confirming that Bpz was the ligand reduced. The other three compounds showed irreversible reductions associated with the tmam ligand. The irreversible nature of this reduction has been shown for similar compounds and ligands.^22,61^ The reduction potential was therefore estimated from the peak cathodic current of the square-wave voltammogram. The excited-state reduction potential (Ru-LL4+*/3+) was estimated through eqn (2), where Δ*G*^0^_ES_ is the Gibbs free energy change from the ground state to the excited state, which was reported previously through a Franck–Condon line-shape analysis.^45^ All electrochemical values are included in Table 2.

2 *E*_o_(Ru-LL4+*/3+) = *E*_o_(**Ru-LL4+/3+**) + Δ*G*_ES_

**Table 2:** Spectroscopic and thermodynamic properties of the **Ru-LL** compounds

**Ru-LL**	**Ru-LL5+/4+** (V)	**Ru-LL4+/3+** (V)	Δ*G*^0^_ES_^a^ (eV)	Ru-LL4+*/3+ (V)	*τ*_0_ (μs)
**Ru-Bpz**	2.10^b^	−0.50^b^	2.09	1.59	1.78
**Ru-Bpy**	1.57	−0.79	2.02	1.23	0.61
**Ru-Dtb**	1.51	−0.80	1.97	1.18	0.36
**Ru-OMe**	1.39	−0.86	1.88	1.02	0.17

a Data from ref. 45.

b Data taken from ref. 44.

**Fig. 4:**
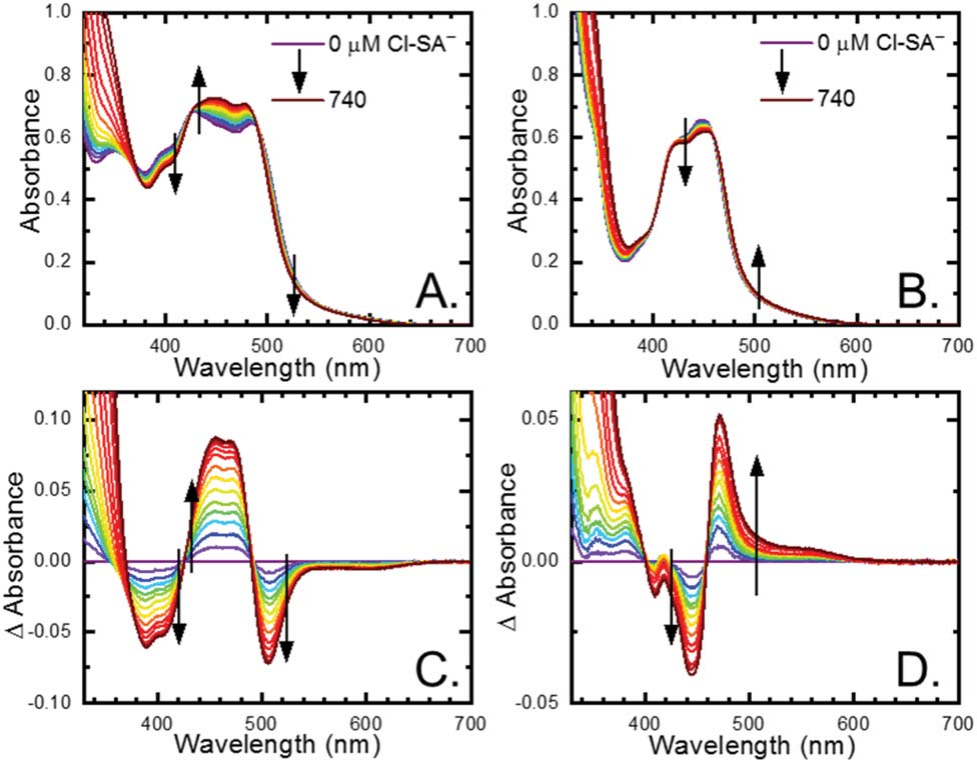
UV-visible absorption spectra of (A) **Ru-Dtb** and (B) **Ru-Bpz** (∼25 μM) upon the addition of 0 to 740 μM **Cl-SA−**. Difference spectra calculated by subtracting the absorption spectra at no **Cl-SA−** from the spectra with **Cl-SA−** present for (C) **Ru-Dtb** and (D) **Ru-Bpz**. Arrows indicate the spectral changes upon **Cl-SA−** addition.

**Fig. 5:**
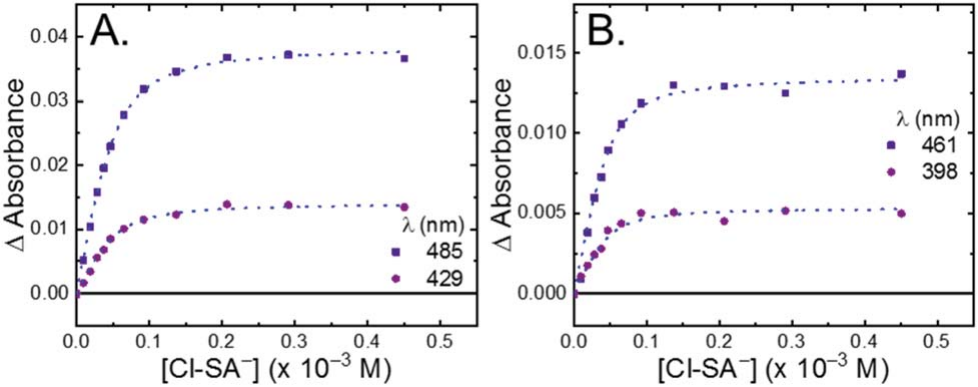
Change in absorbance at the indicated wavelengths from the titration of (A) **Ru-Dtb** and (B) **Ru-Bpz** (25 μM) with **Cl-SA−**. Wavelengths were chosen as they are the isosbestic points for the formation of the doubly ion-paired species and therefore the changes are only due to the first ion-pair formation. The blue dotted line is a fit to a 1:1 binding model.

All four **Ru-LL** compounds exhibited UV-vis absorption spectra with transitions between 200–650 nm. The low energy absorption bands centered around 460 nm were assigned as metal-to-ligand charge-transfer (MLCT) transitions.^62^ Absorption features in the UV were due to ligand centered transitions. The addition of the **R-SA−** derivatives to CH_3_CN solutions of **Ru-LL** induced changes in the UV-vis absorption spectra. Fig. 4 shows a representative example of **Cl-SA−** with **Ru-Dtb** and **Ru-Bpz**. For **Ru-Bpz**, a bathochromic shift in the low-energy MLCT and decrease in the MLCT intensity occurred. For **Ru-Dtb**, **Ru-OMe**, and **Ru-Bpy** a hypsochromic shift of the MLCT was accompanied by an increase in MLCT intensity. Isosbestic points were maintained up to 5 equivalents of added **R-SA−**. These changes were assigned to the formation of the ground-state ion pair, **[Ru-LL4+,R-SA−]3+**. These changes could be well described by a 1:1 binding model,^63^Fig. 5, which provided the equilibrium constant (*K*_EQ,1_), for ion-pair formation, **[Ru-LL4+,R-SA]3+**.^63^*K*_EQ,1_ ranged from 0.5 to 3 × 10^5^ for all 28 combinations of **Ru-LL** and **R-SA−**, Table 3. The association constants measured for these exclusively electrostatic ion pairs, formed in the relatively polar CH_3_CN, are significantly larger than those generally measured in hydrogen bond systems (*K* ≤ 10^3^). These electrostatic ion pairs are also orders of magnitude larger than salt-bridged systems in polar organic solvents (*K* ≈ 10^2^ to 10^3^ in DMSO) and on the higher end of systems reported in nonpolar organic solvents (*K* ≈ 10^4^ to 10^5^ in CH_2_Cl_2_).^31,33–37^

**Table 3:** First ion-pairing equilibrium constants

Compounds	*K*_EQ,1_ (×10^5^)			
	**Ru-Bpz**	**Ru-Bpy**	**Ru-Dtb**	**Ru-OMe**
**OH-SA−**	2.3 ± 0.8	1.4 ± 0.3	2.0 ± 0.2	1.8 ± 0.5
**OMe-SA−**	1.5 ± 0.2	0.8 ± 0.1	1.1 ± 0.1	2.5 ± 0.6
**Me-SA−**	2.4 ± 0.5	1.7 ± 0.2	1.8 ± 0.3	2.4 ± 0.7
**H-SA−**	1.2 ± 0.2	1.2 ± 0.1	1.7 ± 0.2	3.0 ± 0.6
**F-SA−**	0.9 ± 0.3	0.50 ± 0.04	1.1 ± 0.1	1.2 ± 0.1
**Cl-SA−**	1.2 ± 0.2	0.55 ± 0.02	0.9 ± 0.1	1.0 ± 0.1
**Acetyl-SA−**	1.4 ± 0.2	0.83 ± 0.07	2.0 ± 0.7	1.2 ± 0.1

The isosbestic points shifted slightly, by <5 nm, upon **R-SA−** additions larger than 5 equivalents for many of the combinations. This shift indicated that a second **R-SA−** bound to the ion pair, **[Ru-LL4+,2R-SA−]2+**. A 1:1 binding model was used to fit the changes in the absorbance monitored at the first set of isosbestic points. This allowed the changes in absorbance associated with the second ion pairing to be estimated. This analysis yielded second equilibrium constants (*K*_EQ,2_) of 1–5 × 10^3^; about two orders of magnitude lower than the first ion pair. The doubly ion-paired species will not be discussed further as the concentrations utilized to study the ES-PCET reaction were not large enough for an appreciable amount to form.

**Fig. 6:**
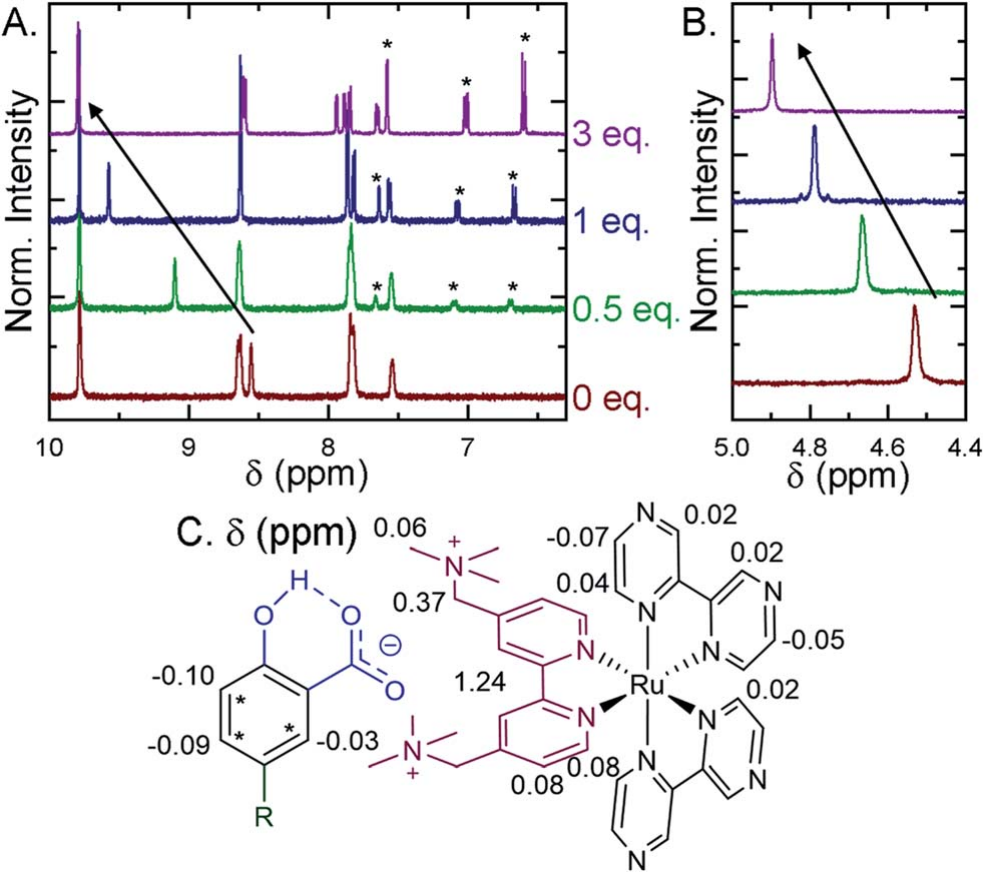
^1^H Nuclear magnetic resonance spectra recorded in CD_3_CN for **Ru-Bpz** upon the addition of up to 3 eq. of **Me-SA−**. (A) Shows the aromatic region, asterisks mark the proton resonances of **Me-SA−**. (B) Shows the methylene resonances on the tmam ligand. Arrows show the downfield shift of the (A) 3,3′-tmam protons and (B) the methylene tmam protons. (C) The total change in chemical shift (Δppm) between 0 and 3 eq. of **Me-SA−** shown for all proton resonances on **Ru-Bpz** and **Me-SA−**.

Selected ion pairs were characterized by ^1^H nuclear magnetic resonance (NMR). The addition of 0.5, 1, and 3 eq. of **Me-SA−** to CD_3_CN solutions of **Ru-Bpz** and **Ru-Dtb** led to measurable shifts in the resonances of the protons on the polypyridyl ligands, Fig. 6. Large downfield shifts were observed only in the resonances of the 3,3′- and CH_2_-protons on the dicationic tmam ligand, Fig. 6C. No significant shifts were detected for the proton resonances of the Dtb or Bpz ligands. This confirmed that the ion pair was formed through interaction of the **R-SA−** with the dicationic tmam ligand. Small upfield shifts were also quantified for the **Me-SA−** aromatic protons.

### Proton-coupled electron transfer excited-state quenching

Pulsed laser excitation of the **Ru-LL** compounds in CH_3_CN led to room temperature, visible photoluminescence from the ^3^MLCT excited state (Ru-LL4+*). With no **R-SA−** present, exponential decays were fit to a first-order kinetic model. The excited-state lifetimes (*τ*) decreased with the electron donating ability of the ancillary bipyridine ligands, **Ru-OMe** < **Ru-Dtb** < **Ru-Bpy** < **Ru-Bpz**, Table 2.

**Fig. 7:**
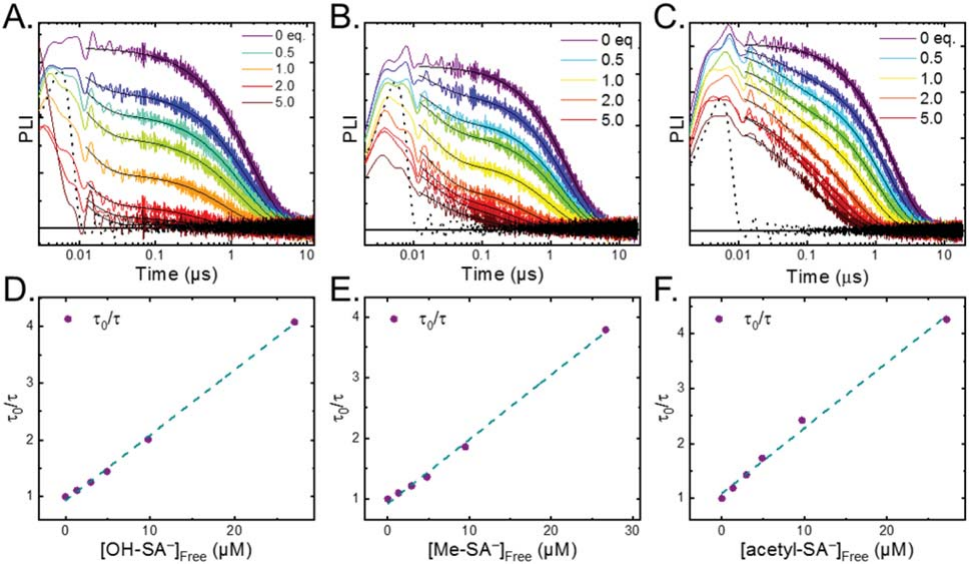
A–C) Time-resolved photoluminescence decays for **Ru-Bpz** (25 μM) upon the addition of up to 5 equivalents of (A and D) **OH-SA−**, (B and E) **Me-SA−**, (C and F) **acetyl-SA−**. Black dotted lines are the instrument response function based on a scattered laser pulse with no sample present. (D–F) Dynamic Stern–Volmer plots. Blue dashed line is a linear fit to the data.

The addition of salicylate anions to solutions of **Ru-LL** quenched the excited-state photoluminescence in 13 of the 28 **[Ru-LL4+,R-SA−]** combinations. These are listed in Table 4. The other combinations did not show excited-state quenching due to unfavorable driving forces (Δ*G*^0^ > 0) and short-lived excited-state lifetimes. For **Ru-Bpz**, excited-state quenching was observed with all seven **R-SA−** derivatives, Fig. 7. Upon the addition of **R-SA−**, the time-resolved photoluminescence decays could not be modelled as first-order decays. Instead, a sum of two exponential decays (biexponential) was needed, eqn (3). The longer lifetime, *τ*_d_, decreased with increased **R-SA−** concentration. A Stern–Volmer analysis,^64,65^Fig. 7, of the lifetimes (*τ*_o_/*τ*_d_) was linear *vs.* the free concentration of **R-SA−** and gave a Stern–Volmer constant (*K*_SV_) of around 1.1 × 10^5^ M^−1^ for **Ru-Bpz**, eqn (4). *K*_SV_ is related to the bimolecular quenching rate constant (*k*_q_) by the lifetime (*τ*_o_) of the excited state in the absence of quencher (*τ*_d,0_): *K*_SV_ = *k*_q_*τ*_d,0_. The quenching rate constant was thus calculated to be *k*_q_ ∼ 6.2 × 10^10^ M^−1^ s^−1^ for all seven **R-SA−** derivatives, Table 4. This is close to the diffusion-limited rate constant for electron-transfer quenching of similar ruthenium complexes by iodide, 6.6 × 10^10^ M^−1^ s^−1^.^66,67^ Therefore, *τ*_d_ was assigned as the lifetime of the diffusional quenching reaction between non-ion-paired **Ru-Bpz** and **R-SA−**.

3 PLI_*t*_ = PLI_1_e^−*t*/*τ*_PCET_^ + PLI_2_e^−*t*/*τ*_d_^

4
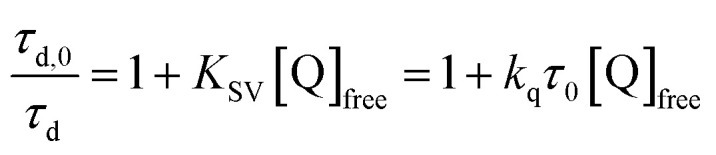


**Table 4:** Excited-state reduction potentials, thermodynamic driving forces, diffusional quenching rate constants, and ion-paired ES-PCET lifetimes and rate constants

**Ru-LL**	**R-SA−**	Δ*G*^0^_PCET_ (eV)	Δ*G*^0^_ET,b_ (eV)	*K*_SV_ (×10^5^ M^−1^)	*k*_q_ (×10^10^ M^−1^ s^−1^)	*τ*_PCET_ (×10^−8^ s)	*k*_PCET_ (×10^7^ s^−1^)	*k*_ET,b_ (×10^7^ s^−1^)
**Ru-Bpz**	OH	−1.03	−1.12	1.15 ± 0.05	6.4	0.8	13	43
	OMe	−0.80	−0.88	1.30 ± 0.04	7.3	0.9	11	28
	Me	−0.62	−0.69	1.06 ± 0.06	5.9	1.4	7.1	10
	H	−0.49	−0.55	1.11 ± 0.05	6.2	2.1	4.8	4.8
	F	−0.48	−0.55	1.11 ± 0.03	6.2	2.4	4.2	6.0
	Cl	−0.44	−0.50	1.20 ± 0.02	6.7	2.6	3.8	3.5
	Acetyl	−0.29	−0.29	1.20 ± 0.04	6.7	4.4	2.3	0.2
**Ru-Bpy**	OH	−0.67	−0.76	0.07 ± 0.01	1.1	1.9	5.3	18
	OMe	−0.44	−0.52	0.10 ± 0.01	1.6	2.9	3.4	8.6
	Me	−0.26	−0.33	a	a	8.6	1.2	1.7
**Ru-Dtb**	OH	−0.62	−0.71	a	a	2.1	4.7	15
	OMe	−0.39	−0.47	a	a	4.5	2.2	5.6
**Ru-OMe**	OH	−0.46	−0.55	a	a	1.6	6.3	21

a Dynamic quenching not observed over the range of concentrations studied.

The shorter lifetime of the biexponential model was independent of the concentration of **R-SA−** and could be fixed throughout the titration. This lifetime varied from 8 ns for **Ru-Bpz** with **OH-SA−** to 86 ns for **Ru-Bpy** with **Me-SA−**, Table 4. The concentration independence of the rate constants indicated that the photoluminescence quenching occurred within the ion pair, and not from a diffusional reaction. This lifetime was assigned to ES-PCET within the ion pair, (*τ*_PCET_), where 1/*τ*_PCET_ provided the rate constant for ES-PCET (*k*_PCET_).

Due to the shorter intrinsic lifetimes of **Ru-Dtb** and **Ru-OMe**, diffusional quenching was too slow to be detected at the concentrations utilized. A biexponential was still needed to model the excited-state relaxation in the presence of **OH-SA−**. The short lifetime still corresponded to the PCET lifetime, and the longer lifetime could be fixed to the lifetime of the complex without quencher.

**Fig. 8:**
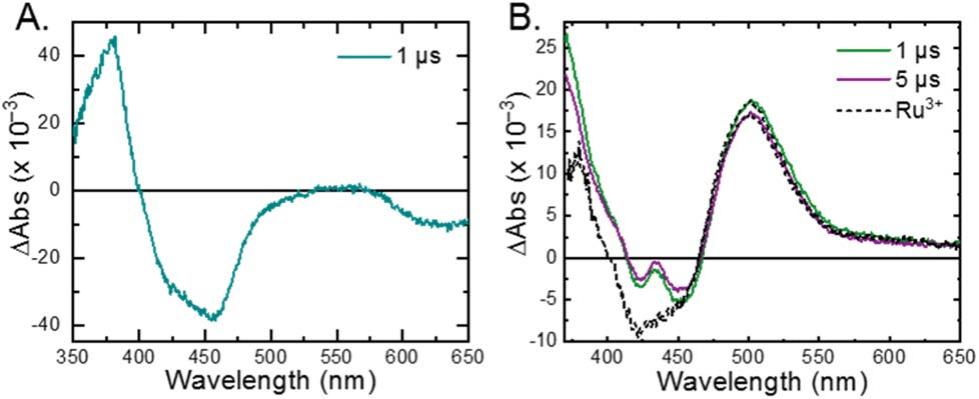
(A) Excited-state transient absorption spectrum of Ru-Bpz4+* obtained 1 μs after laser excitation. The negative change in absorbance at wavelengths longer than 570 nm is from uncorrected emission. (B) Transient absorption spectra collected 1 and 5 μs after laser excitation of Ru-Bpz4+* (25 μM) in the presence of **Cl-SA−** (75 μM). Overlaid is the normalized **Ru-Bpz3+** delta absorbance spectra. Deviation from the **Ru3+** spectra is due to the absorbance of **R-SA˙O**.

The ^3^MLCT excited state of **Ru-Bpz** was produced for nanosecond transient absorption spectroscopy through pulsed laser excitation. In ruthenium polypyridyl excited states the electron resides on the most electron withdrawing ligand.^68^ Therefore, for **Ru-Bpz**, the excited state can formally be described as an oxidized Ru^III^ metal center with a reduced Bpz ligand, [Ru^III^(tmam)(Bpz)(Bpz^−^)]^4+*^, Ru-Bpz4+*. The appearance of absorption features that correspond to the reduced ligand, a positive delta absorbance at ∼380 nm, and the loss of the ground-state MLCT, a negative delta absorbance at 450 nm, were indicative of the excited state, Fig. 8A. These features decayed to the ground-state with an identical lifetime to that of the time-resolved photoluminescence. In the case of **Ru-Bpy**, **Ru-Dtb**, and **Ru-OMe**, the localization of the electron in the MLCT excited state is expected to localize on the quaternary amine ligand (tmam) and thus the excited state can be formally described as [Ru^III^(tmam^−^)(LL)_2_]^4+*^, where LL is Bpy, Dtb, or OMe. This change in excited-state electron localization has been previously proposed in these and similar compounds.^44,45^

**Scheme 3:**
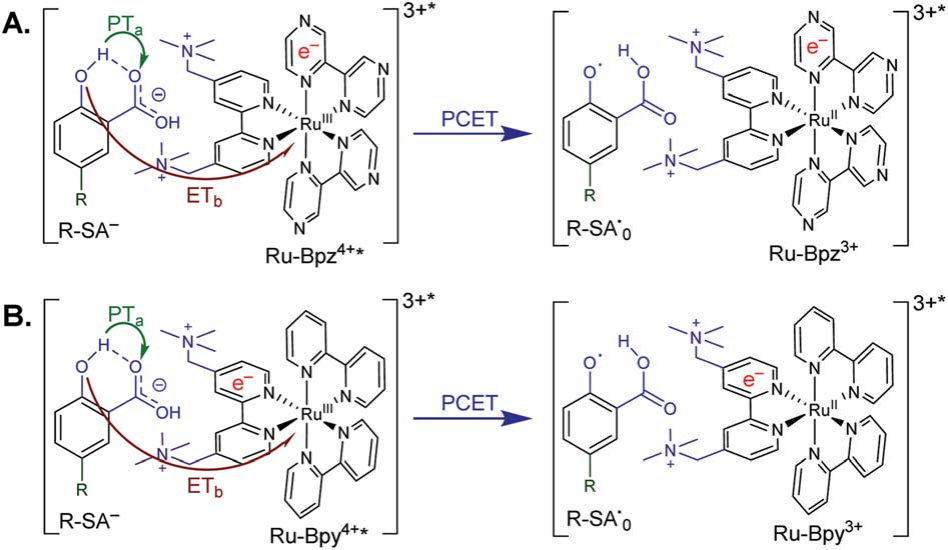
Generic mechanism for the ES-PCET reaction within the [Ru-LL4+*,R-SA−]3+* ion pair, (A) **Ru-Bpz** and (B) **Ru-Bpy**. Green arrow shows the proton transfer and red arrow shows the electron transfer.

Excitation of a solution of **Ru-LL** in the presence of **R-SA−** gave significant differences in the nanosecond transient absorption spectra. Fig. 8B shows representative spectra collected 1 and 5 μs after excitation of the **[Ru-Bpz4+,Cl-SA−]3+** ion pair. The appearance of an absorption band centered around 510 nm was consistent with the formation of the reduced ruthenium complex ([Ru^II^(tmam)(bpz)(bpz^−^)]^3+^, **Ru-Bpz3+**). To confirm this, the **Ru-Bpz3+** delta absorption spectrum was generated through reductive excited-state quenching by tri-*p*-tolylamine. This spectrum could be normalized to the spectra of the reduced ion pair at lower energy wavelengths (>450 nm). A positive deviation from the reduced complex spectra was present at higher energies, around 430 nm, Fig. 8B. It has been reported that an oxidized phenoxyl radical (PhO˙) absorbs light in this region, *e.g.* the tyrosine phenoxyl radical has an absorption at 410 nm.^69–71^ Therefore, this absorption was assigned to the oxidized salicylate, in which the proton has transferred to the carboxylate group, **R-SA˙O**, Scheme 3. This provided a clear indication that the excited state was quenched by an ES-PCET reaction. Similar spectral features were obtained for all **[Ru-LL4+,R-SA−]3+** ion pairs that showed photoluminescence quenching.

**Fig. 9:**
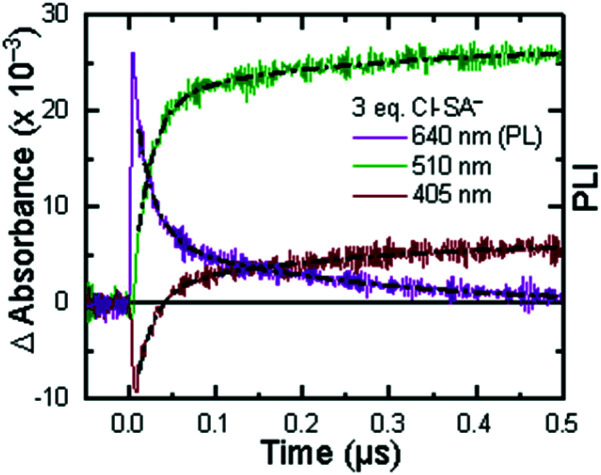
Single wavelength transient absorption kinetics monitored at 405 nm, red, and 510 nm, green, for **[Ru-LL4+,Cl-SA−]3+**. The purple trace is the excited-state decay. Overlaid in black are biexponential fits with the two lifetimes shared between the three traces. The negative signal is due to small amounts of excited-state bleach still present of the monitored wavelengths.

Single wavelength kinetic analysis at wavelengths near the isosbestic points between the ground and excited state of **Ru-Bpz** (405 nm and 510 nm) in the presence of **R-SA−** allowed the formation of **Ru-LL3+** (510 nm) and **R-SA˙O** (405 nm) to be monitored independent of the excited-state decay, Fig. 9. The emission decay was also monitored to directly compare the rates of formation with the excited-state decay. The absorption changes both at 405 nm and 510 nm showed a biexponential signal rise and yielded lifetimes that agreed with the time-resolved photoluminescence titrations, Fig. 9. In fact, the ratio of the pre-exponential factors for the two lifetimes, A_PCET_/A_d_, aligned with those of the excited-state decay. This ratio also aligned with the expected ratio of free ruthenium complex to ion-paired ruthenium, **Ru-LLfree**:**[Ru-LL4+,R-SA−]3+**, based on the equilibrium constant for ion-pair formation, *K*_EQ,1_. This indicated that quenching through both the diffusional reaction and from the pre-formed ion pair occurred through ES-PCET. Both the oxidized salicylate radical and the reduced ruthenium complex could be identified as primary photoproducts, as both **R-SA˙O** and **Ru-LL3+** had identical formation rate constants that aligned with excited-state decay.

Cage escape quantum yields (*Ø*_CE_) of the photoproducts were estimated at 3 eq. of the respective **R-SA−** with **Ru-Bpz**. The change in absorbance at 510 nm, which corresponds solely to the reduced ruthenium complex, was used to estimate the concentration of cage escaped products and the unity internal conversion efficiency of Ru(bpy)_3_^2+^ used as an actinometer. Quantum yields of 0.60–0.70 were calculated for all **R-SA−** derivatives. These cage escape yields are significantly larger than those found in the diffusional excited-state electron-transfer oxidation of iodide by ruthenium excited states (<0.10)^66,72^ and larger than ion-pairs formed between iodide and ruthenium polypyridyl complexes with cape yields of 0.34^73^ and 0.25–0.50.^21^ For PCET in hydrogen-bonded systems, *Ø*_CE_ is often not measured, or irrelevant because the products never dissociate. In cases where measured, *Ø*_CE_ has been much lower than here, see *e.g.*ref. 74. The larger cage escape quantum yields measured here can be rationalized based on changes in coulombic forces in tandem with the proton motion coupled with electron transfer. Upon oxidation, the salicylate derivatives become neutral, which removes the coulombic attraction of the ion-pair. Secondly, unlike simple single-electron-transfer systems, the oxidation of salicylate involves the movement of a proton from the phenolic-OH to the carboxylate functional group. Thus, recombination must also involve the movement of both the proton and electron. The driving force for this proton-coupled back-electron transfer would necessitate considerations of the driving force for both the electron transfer and proton transfer, which may slow the back-reaction rate constant allowing cage escape to compete with and surpass recombination within the solvent cage.

### Mechanistic discussion

The oxidation of salicylate by photooxidized ruthenium complexes is known to occur through a PCET reaction in water.^19,75^ However, to the best of our knowledge, no studies to date have investigated the oxidation mechanism of salicylate in organic solvents. Above, it was shown that the excited state of cationic **Ru-LL** compounds could oxidize salicylate in CH_3_CN. Nanosecond transient absorption spectroscopy confirmed that the reaction proceeded through ES-PCET. Time-resolved photoluminescence experiments showed that the ES-PCET reaction occurred through both a diffusional reaction between non-associated pairs and within the photoexcited ion pairs. While the diffusional reaction occurred near the diffusion limit, 6 × 10^10^ M^−1^ s^−1^, preventing mechanistic analysis, within the pre-formed ion pairs the first-order ES-PCET rate constants were measured directly. Below, we analyse these rate constants along with the above results to probe the ES-PCET mechanism.

As stated in the introduction, this ES-PCET mechanism could proceed through either (1) electron transfer-proton transfer, ET_a_PT_b_, (2) proton transfer-electron transfer, PT_a_ET_b_, or (3) concerted electron-proton transfer, CEPT. Previously, the concerted mechanism was identified for the oxidation of salicylate in water through flash-quench transient absorption spectroscopy and was initially expected to be the active mechanism in CH_3_CN.^19,75^

The appearance of a ground-state tautomer in the absorption spectra of **R-SA−** suggested, however, that the salicylate oxidation mechanism in organic solvents may differ from that in water. In acetonitrile, a stepwise proton-transfer, electron-transfer mechanism could potentially be favored. The ground-state proton transfer would allow electron transfer to occur through the phenolate (**R-O−**), which for **H-SA−** is known to have a more negative redox potential (0.77 V *vs.* NHE in water) than the protonated phenol (1.48 V in water).^19^ Hence, there is a significantly larger driving force for electron transfer through the phenolate. This also suggests that a stepwise electron-transfer, proton-transfer reaction is unfavorable, because the driving force for electron transfer from the protonated phenol would be uphill or have a small favorable driving force in the case of **Ru-Bpz**. Therefore, the possibility that the ES-PCET mechanism follows a stepwise proton transfer-electron transfer mechanism was investigated, depicted in Scheme 4.5
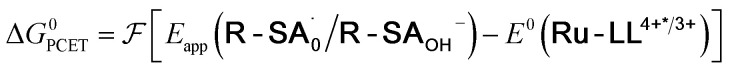
6
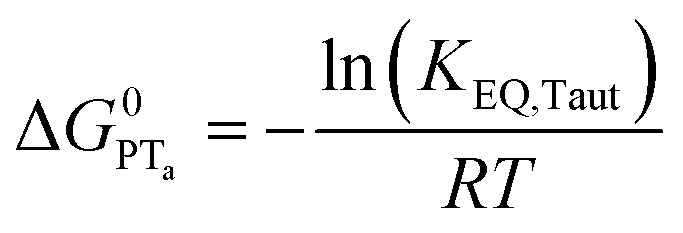
7
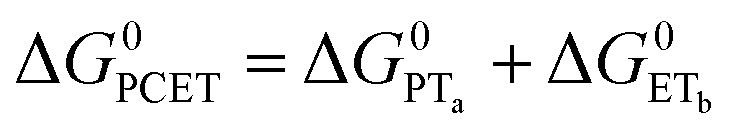
8
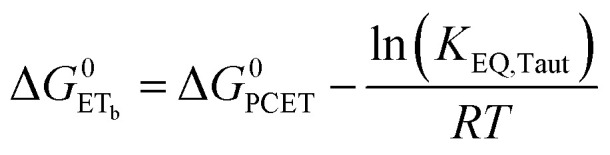
9
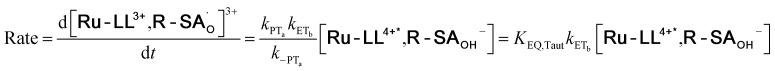
10
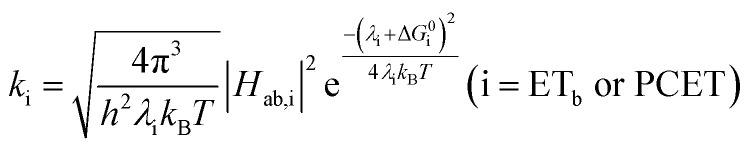

11 Δ*G*° = *N*_A_{*e*[*E*°(D^+^˙/D) − *E*°(A/A^−^˙)] + *ω*(D^+^˙A^−^˙) − *ω*(DA)} − Δ*G*_ES_

12
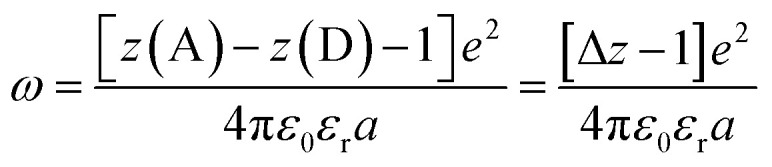


**Scheme 4:**
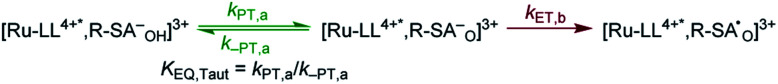
Proposed PTET reaction mechanism within the photoexcited ion pairs.

To start, the Gibbs free energy change for the overall PCET reaction within the ion pair, Δ*G*^0^_PCET_, was estimated through eqn (5),^19^*E*_app_ is the apparent reduction potential for the PCET and encompasses the driving force for proton transfer and the salicylate reduction potential, and is reported in Table 4. This overall driving force can be broken into a sum of the driving force for the initial PT_a_ between the phenolic-OH and carboxylate, Δ*G*^0^_PT,a_, and secondary ET_b_ from the phenolate to the ruthenium excited state, Δ*G*^0^_ET,b_. The estimated tautomerization equilibrium constant, *K*_EQ,Taut_, Table 1, was related to Δ*G*^0^_PT,a_ through eqn (6). Subtraction of Δ*G*^0^_PT,a_ from Δ*G*^0^_PCET_ provided the driving force for the excited-state electron transfer (Δ*G*^0^_ET,b_), eqn (7) and (8). To elucidate the electron-transfer rate constants, the pre-equilibrium approximation was used to develop a rate law based on Scheme 4, eqn (9). Through this assumption, the *k*_PCET_ measured *via* time-resolved photoluminescence is equal to *K*_EQ,Taut_ × *k*_ET,b_, and thus simple division provided *k*_ET,b_.

**Fig. 10:**
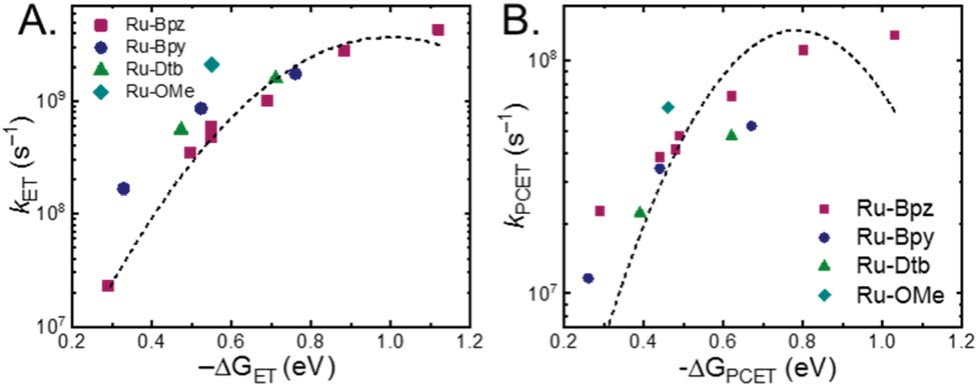
(A) Relationship between the rate constants for ET_b_ within the ion pair and the driving force for electron transfer. (B) Relationship between the observed PCET rate constant and driving force for PCET (note the *Y*-axis spans a much smaller range than in (A)). Dashed lines are best fits to the Marcus equation with a *Y*^2^ weighting restricted to the data points for **Ru-Bpz** (purple, squares). Note, the fit is significantly better for ET over PCET.

A plot of *k*_ET,b_*vs.* Δ*G*_ET_ in Fig. 10A showed the increase in rate constant with increasing driving force for electron transfer in the normal to near barrierless region as described by Marcus theory.^40,76^**Ru-Bpz** provided the most complete data set (7 points) and these data provided a reasonable fit to Marcus theory, eqn (10).^40,43^ The fit, dashed line in Fig. 10A, allowed the two variable parameters of the Marcus equation, *λ* and *H*_ab_, to float. All constants were fixed to their known values and the temperature was fixed as 298 K (room temperature). Thus, the fit is rather well-defined by the curvature and the approximate rate maximum. From this fit a reorganization energy, *λ*, of 1.0 eV (8100 cm^−1^) was approximated. This is close to the expected ∼1.0 eV for electron transfer with ruthenium polypyridyl compounds.^77^ The electronic coupling, *H*_ab_, was estimated as 2.5 × 10^−4^ eV (2 cm^−1^), indicative of a non-adiabatic electron transfer. For comparison, a fit to *k*_PCET_*vs.* −Δ*G*^0^_PCET_ using eqn (10) (i = PCET) resulted in a very poor fit (Fig. 10B); a clear indication that a concerted mechanism was not operational under our conditions. No turnover of the kinetics to a Marcus inverted barrier was discernible. Note that the data are first-order rate constants within the ion pairs that are not limited by diffusion. Nevertheless, the minimal curvature in the plots reduces the accuracy of the fit. This may impact the true values of *λ* and *H*_ab_, but their magnitudes should be reasonable approximations.

The other three ruthenium complexes were also quenched through the same mechanism with the most electron donating **R-SA−** derivatives, **OH-**, **OMe-**, and **Me-**, Fig. 10A. However, the estimated *k*_ET,b_ deviated from the Marcus curve defined by **Ru-Bpz**, with most of the rate constants larger than would be expected. The reason for this deviation is unclear, however we believe it comes about due to the difference in the localization of the excited electron in the photoexcited ruthenium complexes. As shown in Scheme 3, the excited state of **Ru-Bpz** localizes the excited electron on the ancillary Bpz ligand away from the direction of charge transfer. Whereas, for **Ru-Bpy**, **Ru-Dtb**, and **Ru-OMe** the electron is localized on the cationic tmam ligand directly between the bound salicylate and the ruthenium. At a first glance this difference in localization should slow the electron transfer as the electron localized on the tmam ligand would repel the anionic salycilate,^78^ lengthening the electron transfer distance. It may also be expected that electron transfer across a reduced bipyridine ligand (by super-exchange or hopping)^79^ would be less favorable, due to the high energy of the (virtual) intermediate with two electrons added to the ligand. However, facile charge transfer across reduced bipyridine ligands has been observed in the case of iridium^31^ complexes and therefore, we suggest that the orientation of the acceptor Ruthenium d-orbitals relative to the salicylate, which should not depend on the ^3^MLCT localization, are more likely to matter than the charge on the bipyridine ligand.

Another factor that electron localization would affect is the electrostatic work terms, *ω*, for the electron-transfer reaction. These work terms account for the free energy needed to bring the reactants together and to separate the products and should be included in the approximation of the driving force for electron transfer, and through extrapolation a PCET, eqn (11), where D and A are the electron donor and acceptor reactants, *N*_A_ is the fundamental charge, and *e* is the elementary charge.^56,80^ These work terms can be simplified to eqn (12), where *z* is the charge of the reactant, *ε*_0_ is the vacuum permittivity, *ε*_r_ is the solvent dielectric, and *a* is the distance between the donor and acceptor. In this study, the difference in charge is 3+, which means the work term is positive, decreasing the overall driving force for the PCET reaction.

The variation between **Ru-Bpz** and the other complexes may come from the difference in charge distribution in the excited state. For **Ru-Bpy**, **Ru-Dtb**, and **Ru-OMe**, the decrease in positive charge near the salicylate and along the electron-transfer vector due to electron localization on the tmam ligand would lower the magnitude of the work term and increase the overall driving force for the reaction, shifting the data for those three complexes to more negative Δ*G* values in Fig. 10A. The reverse occurs for **Ru-Bpz**. Therefore, the Δ*G*^0^_ET,b_ for **Ru-Bpy**, **Ru-Dtb**, and **Ru-OMe** is underestimated relative to **Ru-Bpz** and the rate constants deviate from the fit. Typically, a spherical approximation of the reactants and products could provide a reasonable estimate for the work terms.^55^ However, for the **Ru-LL** compounds studied this would not differentiate the two excited states. Due to the complexity of charge distribution in the ruthenium complexes and salicylate derivatives, developing a reasonably accurate approximation for the electrostatic work terms is difficult.^46,73,81,82^ However, within a single ruthenium complex, such as **Ru-Bpz**, these work terms are not be expected to vary significantly, and as such we chose to model solely the **Ru-Bpz** data as it provided the largest uniform series, Fig. 10A.

The weak electronic coupling constant measured for the electron-transfer step of the PCET reaction implies a non-adiabatic electron transfer. This is expected for an outer-sphere electron transfer reaction from salicylate to the excited ruthenium complex. In overall non-adiabatic concerted proton-coupled electron transfer reactions, the CEPT coupling constant (*V*_CPET_^2^) is approximately equal to the combination of the electronic coupling and overlap between the proton vibrational wavefunctions, *V*_CPET_^2^ ≈ *V*_ET_^2^ × *S*_PT_.^15^ The present case is extreme to which the electron transfer can be thought of as gated by internal proton transfer within salicylate. Thus, we were able to deconvolute the electron-transfer rate constants, and in turn estimate the electronic coupling constant for electron transfer (*V*_ET_, also referred to as *H*_ab_). This non-adiabaticity of the electron transfer does not indicate that the coupling between the electron and proton is weak.^83–85^ This coupling is intrinsic to the coupling of electron transfer to proton transfer within the salicylate. In studies of the PCET between TEMPOH and carboxylates covalently attached to ruthenium, a CEPT was found even when the electron acceptor and proton acceptor were separated by >10 Å, *i.e.* a very small *V*_ET_^2^.^84,85^ The proton transfer is strongly coupled to electron transfer within TEMPOH and thus, even with little coupling between the carboxylate proton acceptor and ruthenium electron acceptor, the mechanism followed a CEPT. A similar case can be made for the PT_a_ET_b_ mechanism determined for the oxidation of salicylate in acetonitrile. The electron transfer from salicylate is dependent upon the internal proton transfer that reveals a strongly reducing phenolate. Therefore, while the outer-sphere electron transfer to ruthenium is non-adiabatic, the electron transfer is strongly coupled to the internal proton transfer. This is reflected in the large shift of p*K*_a_ upon oxidation of the phenol, and conversely a large shift of *E*^0^ upon deprotonation. Thus, the driving force for CEPT is larger than for the initial steps of ET_a_ and PT_a_, which tends to favor CEPT. On the other hand, CEPT requires tunneling of both electron and proton in the transition state that may have a lower probability than single tunneling of either electron or proton.

The PT_a_ET_b_ oxidation of salicylate reported here comes about due to the appearance of the proton transfer tautomer of the strong internal hydrogen bond. Many groups have studied the oxidation of phenols with internal hydrogen bonds.^84,86^ The ability to tune the structure, proton transfer distance, and hydrogen bond strength make these systems valuable for fundamental studies. We used salicylate as the known, strong internal hydrogen bond was hoped to favor H^+^ tunneling (due to a large *S*_PT_) and potentially a CEPT mechanism, as has been proposed by others under aqueous conditions.^19,20^ However, the present work shows that these strong hydrogen bonds may also favor proton transfer and a PT_a_ET_b_ mechanism. The appearance of a low-energy absorption that was assigned to the ground-state proton-transfer tautomer in the salicylate derivatives studied here aligns with a model proposed by Limbach and co-workers who investigated the same phenomena in *inter*molecular hydrogen bonds between phenols and carboxylates. They defined the “localized charge solvation” concept, which states that “an increase in the solvent polarity induces proton transfer in the sense that charge is transferred toward the acceptor less capable of charge delocalization.”^51^ Furthermore, they state that aprotic, non-polar solvents are better at stabilizing large, delocalized anions such as a phenolate *vs.* small, localized charges such as a carboxylate. Stated another way, the proton localization along the hydrogen bond between the salicylate phenol and carboxylate oxygens is determined by the difference in stabilization energy between the two anionic groups in the solvent of interest. Therefore, while the equilibrium constants measured in acetonitrile were ≤1, if salicylate were to be dissolved in an even more non-polar solvent such as CH_2_Cl_2_, the equilibrium would shift further toward the tautomeric form and the equilibrium constants increase. In the case where no tautomer was observed, water, it was possible to estimate the driving force for *intra*molecular proton transfer through the difference in the p*K*_a_ between the unsubstituted phenol and benzoate (Δ*G*_PT,a_ = +340 mV in water).^18–20^ In acetonitrile this is not the case as the p*K*_a_s measured for the unsubstituted phenol and benzoate do not account for the free energy change associated with the competitive stabilization of the phenol *vs.* the carboxylate within the conjugated salicylate. However, the ability to measure the tautomer equilibrium constant provides an alternative means to access the driving force for proton transfer which was found to be on average an order of magnitude lower than that in water (Δ*G*_PT,a_ +55 mV in acetonitrile for **H-SA−**). This order of magnitude decrease in the proton-transfer driving force is a key factor in the disparate mechanism of salicylate oxidation in acetonitrile *vs.* water.

Work by the Hammarström group on tungsten hydride compounds has detailed a similar change in mechanism with proton-transfer driving force. In the study of tungsten hydrides with an external pyridine base, *i.e.* an *inter*molecular hydrogen bond, the reaction mechanism proceeded through an electron transfer limiting ET_a_PT_b_ mechanism. However, when the same pyridine base was appended to the tungsten hydride to form an *intra*molecular hydrogen bond, the mechanism could proceed as either a CEPT or, in the case of weak oxidants, a pre-equilibrium PT_a_ET_b_ mechanism.^12,87^ Taken together with our present results suggests that while stronger H-bonds may facilitate PT, this does not just favor the CPET mechanism, and instead, enhancing PT also promotes a PT_a_ET_b_ mechanism. This concept is an important principle to fundamental catalyst design where secondary sphere modifications aimed at facilitating proton transfer to favor a concerted reaction pathway must account for the stepwise route.

This work highlights the breadth with which ion pairs may facilitate the study of ES-PCET. Without the need to ensure either the ruthenium complexes or salicylates were covalently connected or had directing hydrogen bonding functionality, 28 combinations of ion pairs could be investigated with 13 providing measurable ES-PCET reactivity. The use of ion pairs facilitated direct measurement of the PCET rate constants and evaluation of the PCET mechanism for salicylate oxidation in organic media. Furthermore, the rate constants for electron transfer within the PCET reaction were found to fall within the normal to near-activation-less region of Marcus' parabola. Most systematic studies of PCET mechanisms have reported rate constants within the linear regime of Marcus theory.^88–92^ Only recently has clear evidence for the Marcus inverted region for concerted PCET been disclosed, in which Mayer and coworkers reported a series of covalently linked donor–acceptor dyads that underwent concerted forward PCET in the normal region of the Marcus parabola and inverted region kinetics for the back reaction.^9,30^ An important feature in these systems was the necessity of covalently linking all three components of the PCET reaction. Ion pairing offers a potential way to remove this limitation while maintaining a first-order reaction. This ability of ion-pairs to reduce the reaction order and overcome diffusion without synthetic difficulty of covalent bonds or need of linked hydrogen bonds has implications not only in fundamental mechanistic studies, but also in applications toward solar fuels^1,4,93,94^ and photosensitized organic synthesis.^8,95^

## Conclusions

In summary, we provide a systematic, spectroscopic ES-PCET mechanistic study that occurs within a photoexcited, coulombic ion pair. These ion pairs formed readily between cationic ruthenium complexes and anionic salicylate derivates in CH_3_CN solution. The use of ion-pairing to preassociate the photosensitizer and salicylate reduced the reaction order from 2 to 1, which provided unimolecular rate constants for the ES-PCET reaction. Kinetic experiments on a series of ruthenium complexes and salicylate derivatives provided a clear curvature in a plot of PCET rate constants *vs.* the driving force for PCET. Correcting for a pre-equilibrium ground-state tautomerization within the salicylate provided electron-transfer rate constants near the Marcus barrierless region, one of the few reported cases where this relation has been found. The ability of ion pairs to reduce reaction orders for complex multicomponent systems has applications throughout chemistry and is commonly used in supramolecular applications. The generality provided by electrostatic interactions has seen limited use in PCET and this study provides a clear extension of this methodology towards fundamental PCET investigations and the ready expansion of this concept to solar fuel and organic photosynthetic applications will be of great interest.

## Conflicts of interest

There are no conflicts to declare.

## Supplementary Material

SC-011-C9SC04941J-s001
