# Supplementary material for: Excited-state proton-coupled electron transfer within ion pairs
Source: Chem Sci. 2020 Mar 3;11(13):3460–73. doi: 10.1039/c9sc04941j (PMC8152629; doi:10.1039/c9sc04941j)
Supplement: SC-011-C9SC04941J-s001 [file SC-011-C9SC04941J-s001.pdf]

Electronic Supplementary Material (ESI) for Chemical Science.
This journal is © The Royal Society of Chemistry 2020

EXCITED-STATE PROTON-COUPLED ELECTRON TRANSFER IN ION PAIRS

Wesley B. Swords, Gerald J. Meyer and Leif Hammarström

Supplementary Information

Reanalysis of Rodgers' Tyrosine-Porphyrin pH Dependent Electron Transfer	2
Salicylate Derivatives Characterization	4
Ruthenium Complexes and Salicylate Ion Pairs	8
Transient Spectroscopy	16
^{1}H NMR	22

Reanalysis of Rodgers' Tyrosine-Porphyrin pH Dependent Electron Transfer

In the 1990s, Rodgers and Aoudia investigated electron transfer between tetracationic Zn and Pd porphyrins and an anionic tetrapeptide terminated in a tyrosine amino acid, Figure S1.[1,2] Thanks to the 4+ and 4– charges on the respective molecules, ion pairs were formed in buffered aqueous solutions. Upon excitation, biexponential kinetics were measured for the porphyrin excited-state decay. The fast lifetime (τ_1) was found to be polypeptide concentration independent and assigned to *electron transfer* within the ion pair. The inverse, $1/\tau_1$, of the fast lifetime provided the rate constant k_1 as 6.6×10^6 s^{-1}. The slower lifetime (τ_2) was found to be concentration dependent and aligned with diffusional quenching by the polypeptide, Figure S2. The Stern–Volmer constant was 6.2×10^9 M^{-1} s^{-1}. Surprisingly, this *electron transfer* was found to be pH dependent. The authors analyze this trend by looking at the log(k_f) vs. pH, extracting a slope of 0.11/pH, Figure 2A. They suggest that this may come from the fact that tyrosine oxidation has a pH dependence of 0.59 V/pH unit, however as this did not align with the measured slope for electron transfer could not make any further insight.[1] In a follow up study they study the temperature dependence of this reaction, again in 0.01 M phosphate buffered solution and estimate a reorganization energy of 1.7 eV was found.[2]

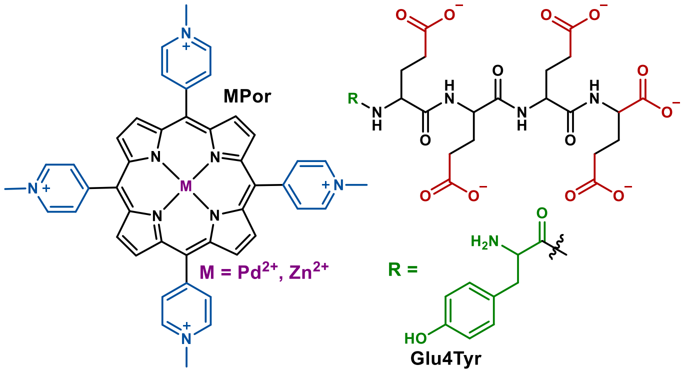

Figure S1. Cationic porphyrins and tyrosine polypeptide used in Rodger's studies.

The one flaw in Rodger's and Aoudia's analysis was that the pH dependence of the electrochemical oxidation of tyrosine is pH dependent because of the proton-coupled electron transfer nature of the oxidation.[3] In theory, the true one-electron oxidation of tyrosine should be completely pH independent, and only when coupled to proton transfer is the 0.59 V/pH unit slope observed. Work after these initial reports by other groups has suggested that oxidation of tyrosine in buffered aqueous solution can occur through a PCET mechanism in which the proton transfers to the base form of the buffer, in this case dibasic phosphate.[4,5] A plot of k_f vs. the dibasic phosphate concentration, Figure 2B, showed a linear relationship with a slope that gave a rate constant for PCET of $k_{PCET,Buffer} = 5.7 \times 10^8$ M^{-1} s^{-1}. In a similar analysis, equation SI1, has been derived from the Henderson-Hasselbalch equation and takes into consideration the fact that PCET can occur to water, k_w. A fit of the reported data to equation SI1, Figure 2C, provided the same $k_{PCET,buffer}$ from Figure 2B and a k_w of 0.5×10^6 s^{-1}. These data, along with literature reported after Rodger's study,[4] provide compelling evidence that the reported excited state reaction proceeds primarily through a PCET reaction.

$$k_f = k_w + f_b(1 + 10^{pKa(HPO_4^{2-})-pH})k_{PCET,Buffer} \quad \text{Equation SI1}$$

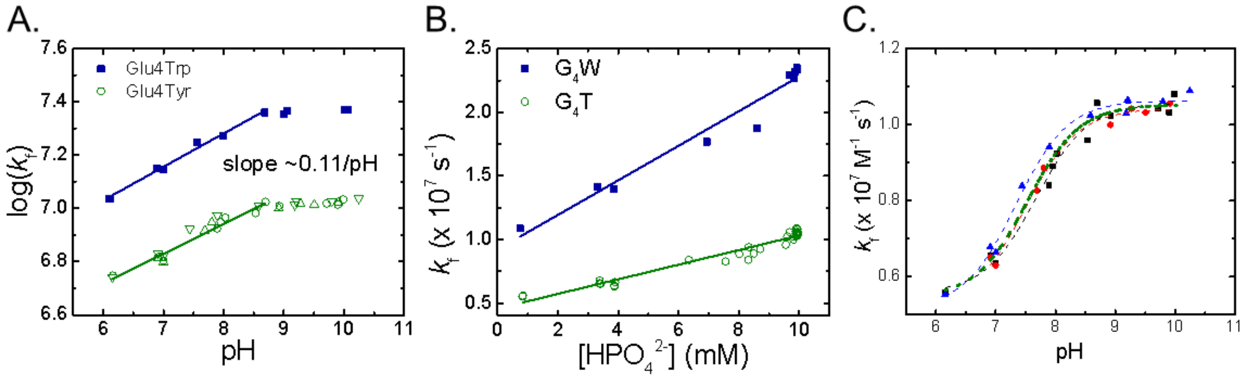

Figure S2. A) pH dependence of log(k_f) in 0.01 M phosphate buffer. Slope of 0.11/pH shown. B) Linear relation of k_f with concentration of dibasic phosphate. Bimolecular rate constant of $k_{PCET,buffer}$ = 5.7 × 10^8 M^{-1} s^{-1} determined from the slope. C) pH dependence of k_f in 0.01 M phosphate buffer. Three experiments were performed by the authors at varying porphyrin concentrations, noted by the colors. Dotted lines are fits to equation SI1 with the green, bold line an average through all. The extracted $k_{PCET,buffer}$ = 5.7 × 10^8 M^{-1} s^{-1} aligned with the analysis vs. the dibasic phosphorous concentration in B. The rate constant for PCET with water was extracted as k_w = 0.5 × 10^6 s^{-1}.

Salicylate Derivatives: Spectroscopy, pK_a determination, tautomerization, electrochemistry

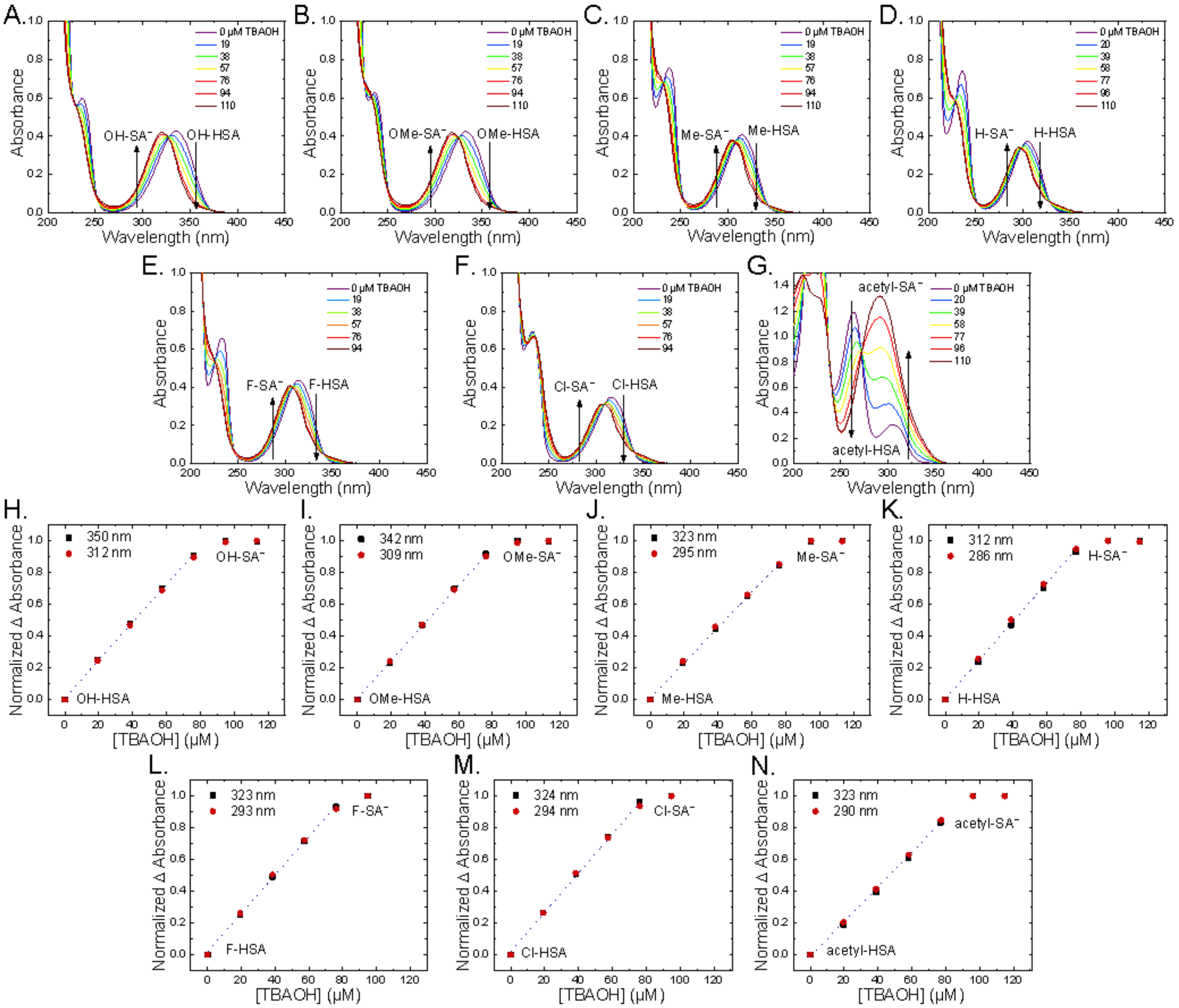

Figure S3. A–G: Titration of R-HSAs (~100 µM in CH_3CN) with the indicated concentrations of TBAOH monitored by UV-Vis spectroscopy. Arrows indicate the direction of change with increased TBAOH concentration for the species noted. H–N: Normalized change in absorption with increased concentration of TBAOH monitored at the indicated wavelengths. A linear regression through the first five additions (up to just under 1 equivalents) is shown (dotted line). The species at the beginning and end of titration are noted. A,H: OH-HSA/SA⁻, B,I: OMe-HSA/SA⁻, C,J: Me-HSA/SA⁻, D,K: H-HSA/SA⁻, E,L: F-HSA/SA⁻, F,M: Cl-HSA/SA⁻, and G,N: acetyl-HSA/SA⁻.

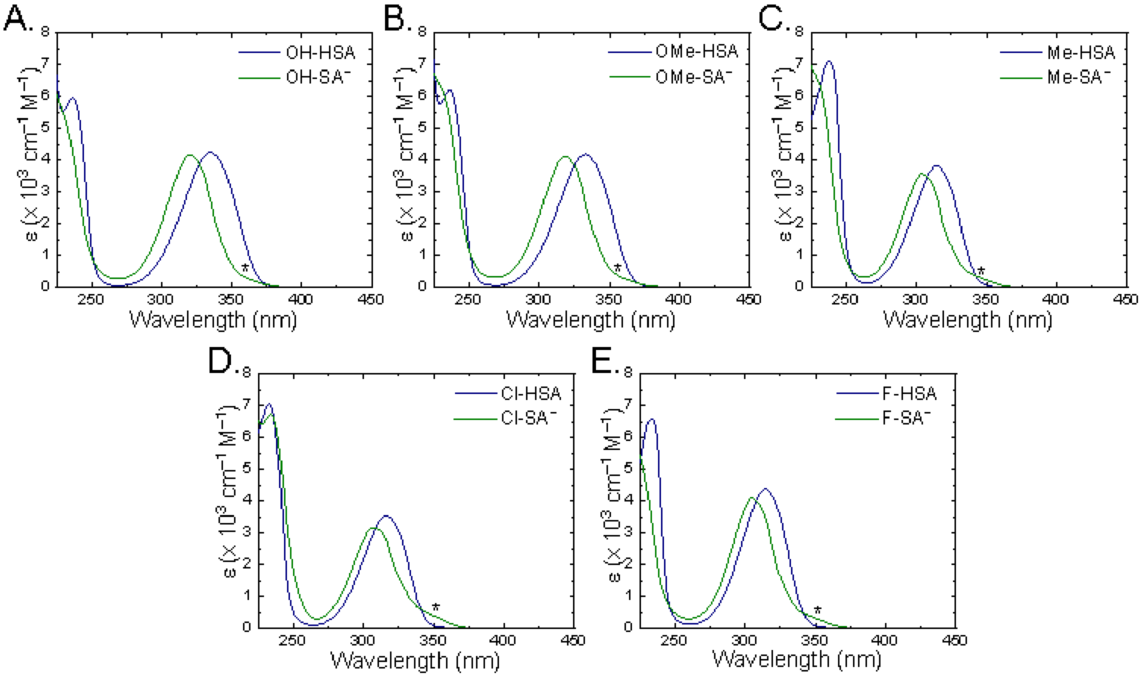

Figure S4. Extinction coefficient spectra for A. OH-HSA/SA^-, B. OMe-HSA/SA^-, C. Me-HSA/SA^-, D. F-HSA/SA^-, and E. Cl-HSA/SA^-. For spectra for H- and acetyl-HSA/SA^- see main text Figure 1A and B respectively. The asterisk (*****) denotes the shoulder corresponding to the SA^- tautomer (see *discussion* in main text).

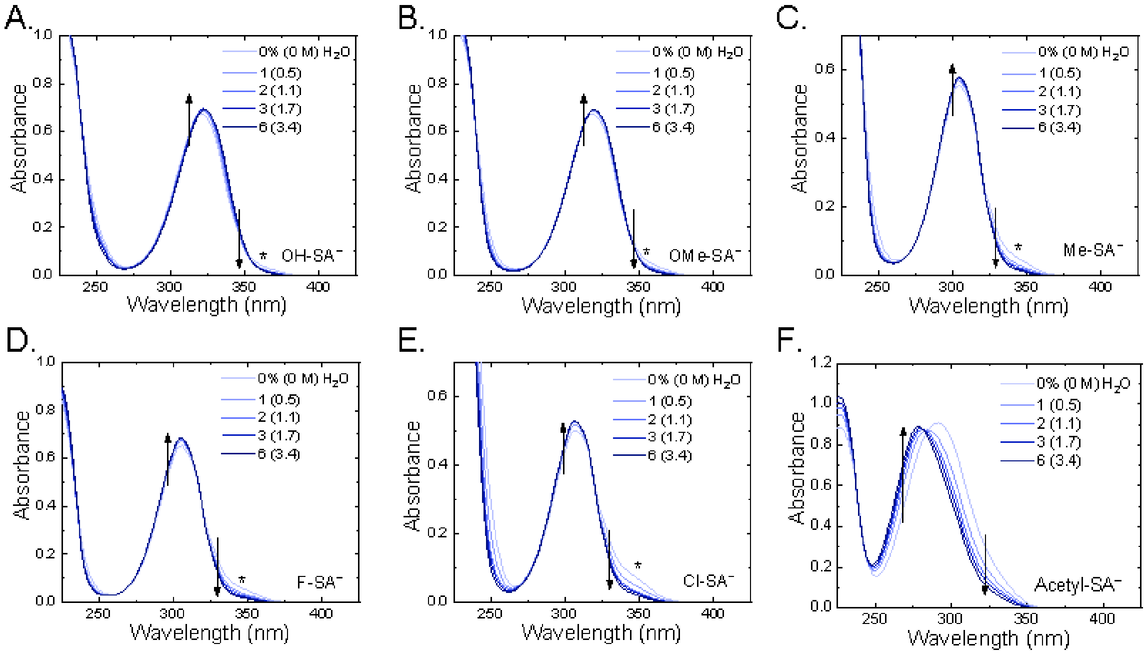

Figure S5. Changes in the UV-Visible absorption spectra associated with the addition of the indicated amounts of water into ~160 μM CH_3CN solutions (66 μM for acetyl-SA^-) of the indicated R-SA^- compounds. Arrows indicate direction of change upon the addition of water. Asterisks (*) indicate the absorption of the tautomeric form.

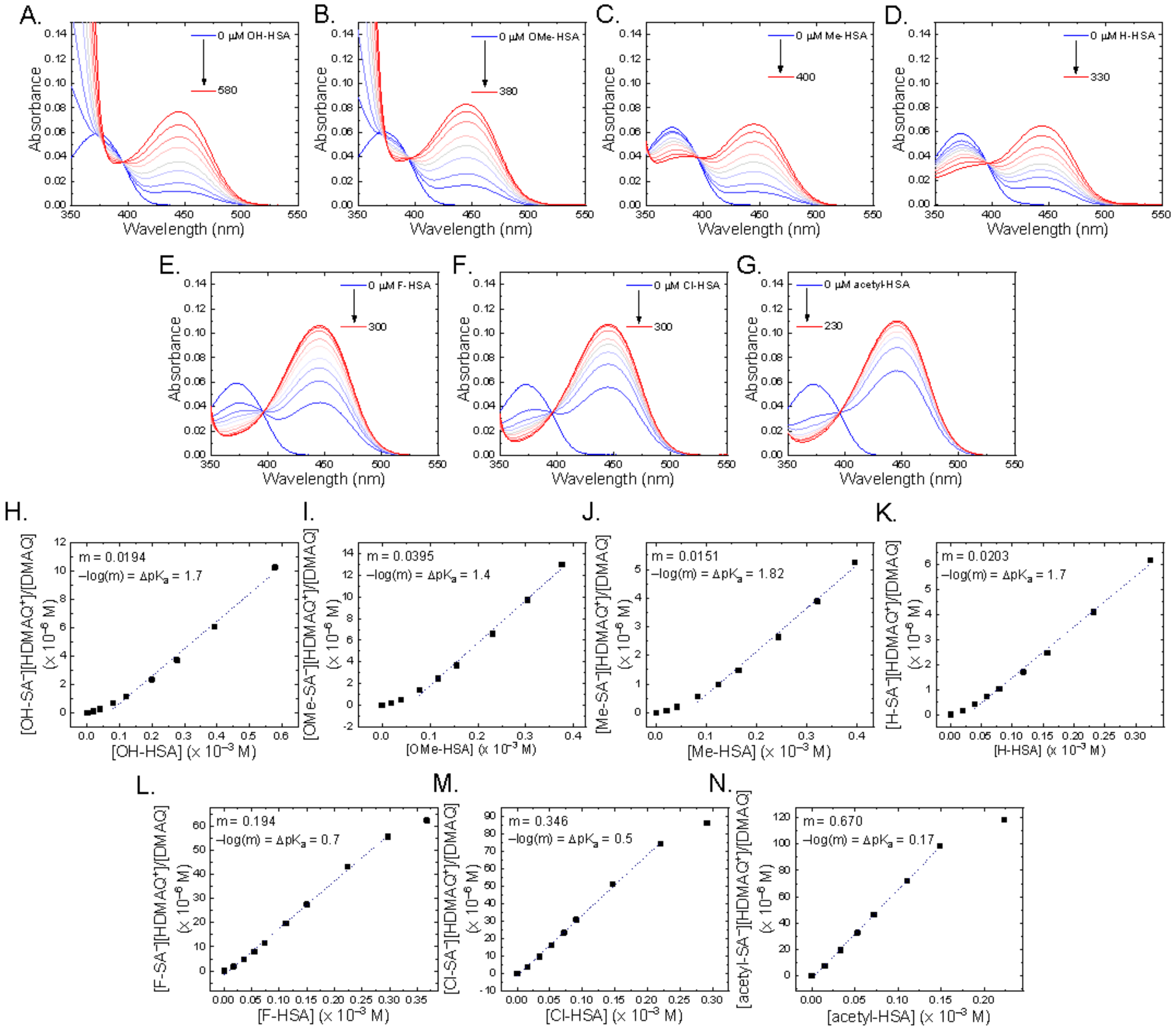

Figure S6. A.–G. Spectrophotometric titrations of TMP-DMAQ (6-8 μM) in CH_3CN with each R-HSA. A. OH-HSA, B. OMe-HSA, C. Me-HSA, D. H-HSA, E. F-HSA, F. Cl-HSA, and G. Acetyl-HSA. H.–N. Rearranging the acid-base equilibrium equation (K_{EQ} = [R-SA$^-$][HDMAQ$^+$]/[R-HSA][DMAQ]) allowed the difference in pK_a versus the known pK_a of TMP-DMAQ (pK_a = 15.2) to be calculated. pK_a values are reported in Table 1 of the main text.

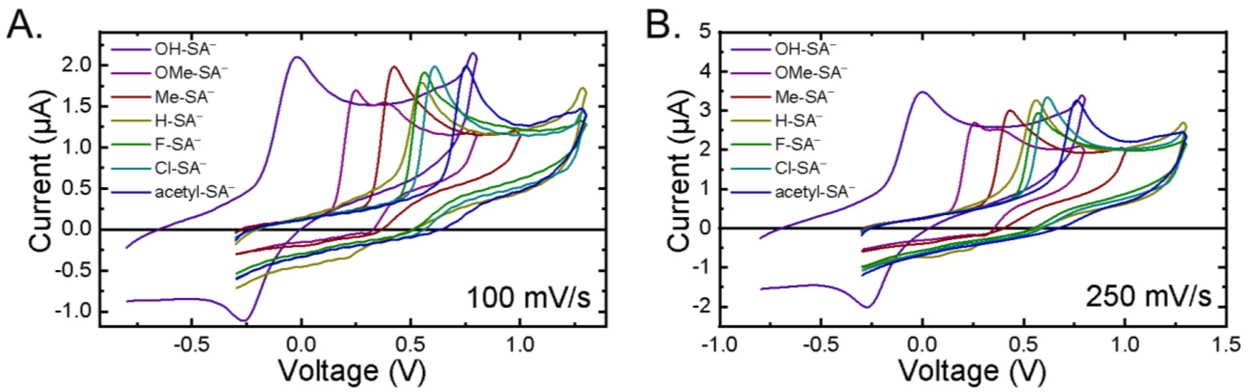

Figure S7. Cyclic voltammograms of R-SA⁻s in 0.1 M $TBAClO_4/CH_3CN$. Scan rates shown are 100 mV/s (A.) and 250 mV/s (B.).

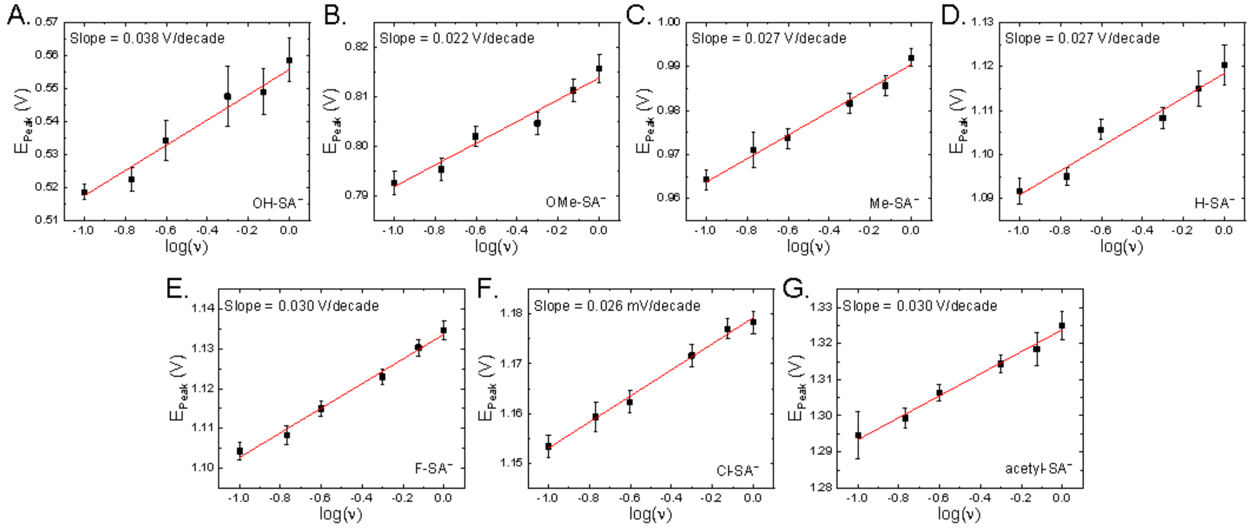

Figure S8. Close up of slopes for scan rate dependence of CVs. Peak voltage plotted vs. the log of the scan rate. Error bars are from three trials with the best fit regression line shown.

Ruthenium complexes and salicylate ion pairs:

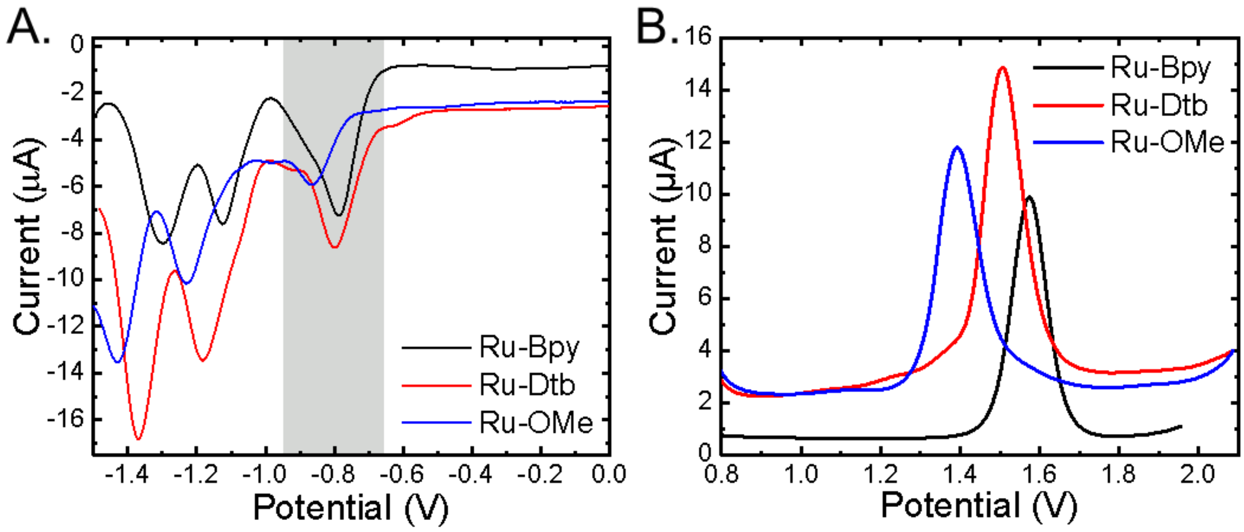

Figure S9. Square-wave voltammetry in 0.1 M $TBAClO_4/CH_3CN$ solution of **Ru-Dtb**, **Ru-Bpy**, **Ru-OMe**. Gray area shows the irreversible reduction of the ruthenium bound tmam ligand.

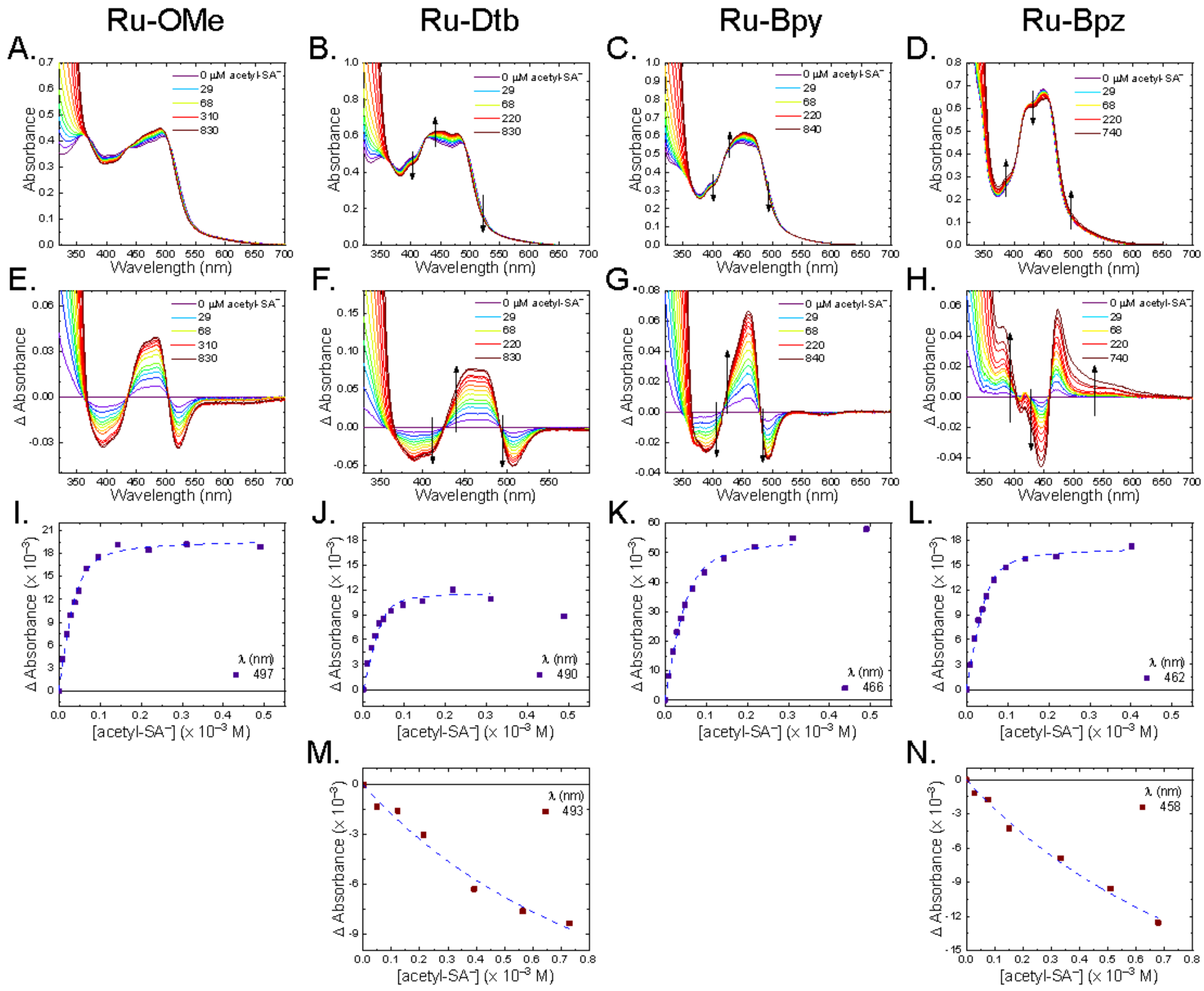

Figure S10. A.–D. UV-Vis absorption titrations of A. **Ru-OMe** (40 µM), B. **Ru-Dtb** (50 µM), C. **Ru-Bpy** (50 µM), and D. **Ru-Bpz** (50 µM) with the indicated acetyl-SA^- concentrations in CH_3CN solutions. E.–H. Show the change in absorption for E. **Ru-OMe**, F. **Ru-Dtb**, G. **Ru-Bpy**, and H. **Ru-Bpz** in which the initial spectra with 0 µM acetyl-SA^- was subtracted from the other spectra. Binding isotherms for the 1st ion pairing are shown, I. **Ru-OMe**, J. **Ru-Dtb**, K. **Ru-Bpy**, and L. **Ru-Bpz**; dashed lines are fits using a 1:1 binding isotherm.[6] A second ion pairing was modeled for both **Ru-Dtb** (M.) and **Ru-Bpz** (N.).

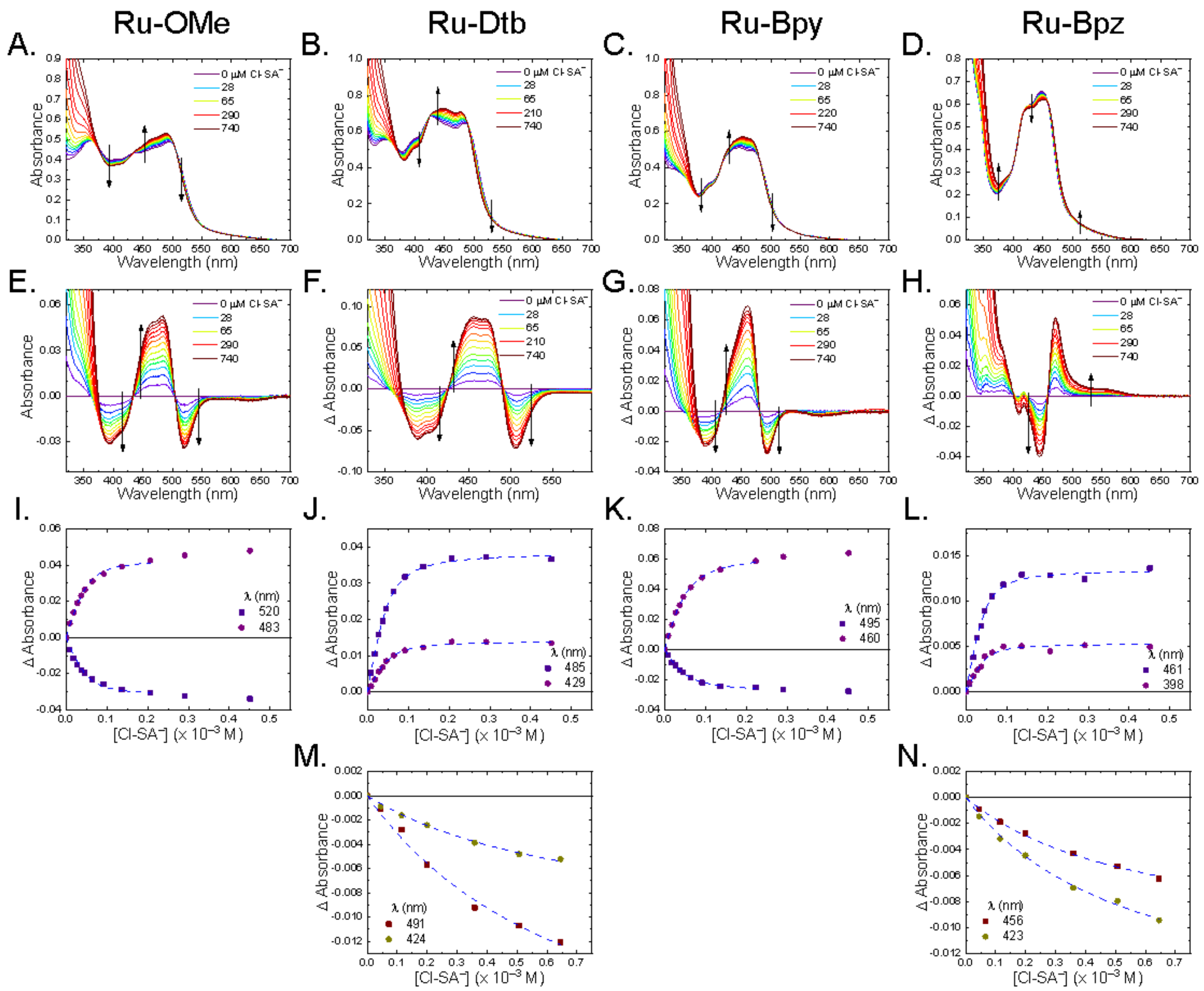

Figure S11. A.–D. UV-Vis absorption titrations of A. **Ru-OMe** (40 μM), B. **Ru-Dtb** (50 μM), C. **Ru-Bpy** (50 μM), and D. **Ru-Bpz** (50 μM) with the indicated Cl-SA⁻ concentrations in CH_3CN solutions. E.–H. Show the change in absorption for E. **Ru-OMe**, F. **Ru-Dtb**, G. **Ru-Bpy**, and H. **Ru-Bpz** in which the initial spectra with 0 μM Cl-SA⁻ was subtracted from the other spectra. Binding isotherms for the 1st ion pairing are shown, I. **Ru-OMe**, J. **Ru-Dtb**, K. **Ru-Bpy**, and L. **Ru-Bpz**; dashed lines are fits using a 1:1 binding isotherm.[6] A second ion pairing was modeled for both **Ru-Dtb** (M.) and **Ru-Bpz** (N.).

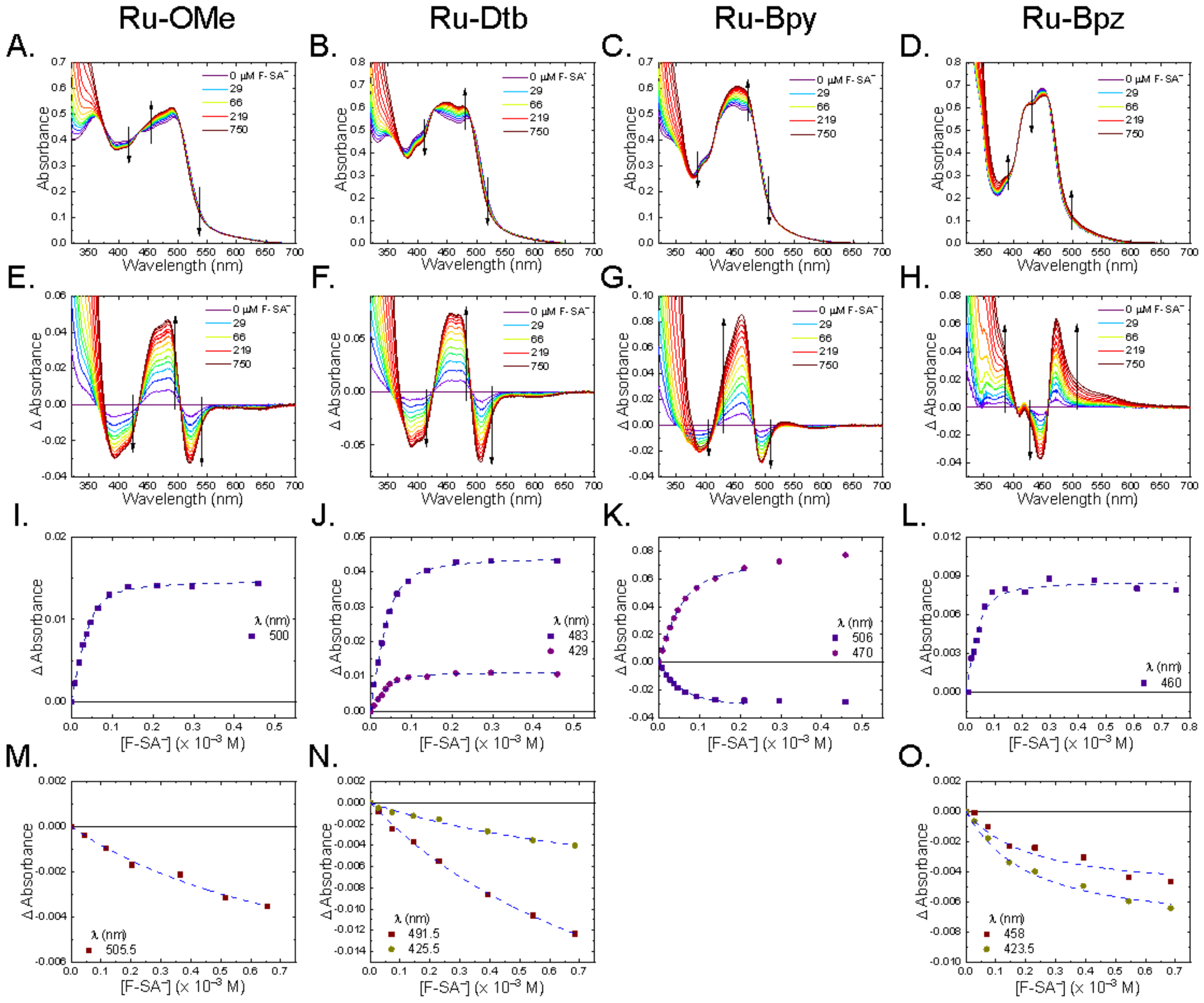

Figure S12. A.–D. UV-Vis absorption titrations of A. **Ru-OMe** (40 µM), B. **Ru-Dtb** (50 µM), C. **Ru-Bpy** (50 µM), and D. **Ru-Bpz** (50 µM) with the indicated F-SA⁻ concentrations in CH_3CN solutions. E.–H. Show the change in absorption for E. **Ru-OMe**, F. **Ru-Dtb**, G. **Ru-Bpy**, and H. **Ru-Bpz** in which the initial spectra with 0 µM F-SA⁻ was subtracted from the other spectra. Binding isotherms for the 1st ion pairing are shown, I. **Ru-OMe**, J. **Ru-Dtb**, K. **Ru-Bpy**, and L. **Ru-Bpz**; dashed lines are fits using a 1:1 binding isotherm.[6] A second ion pairing was modeled for both **Ru-OMe** (M.), **Ru-Dtb** (N.) and **Ru-Bpz** (O.).

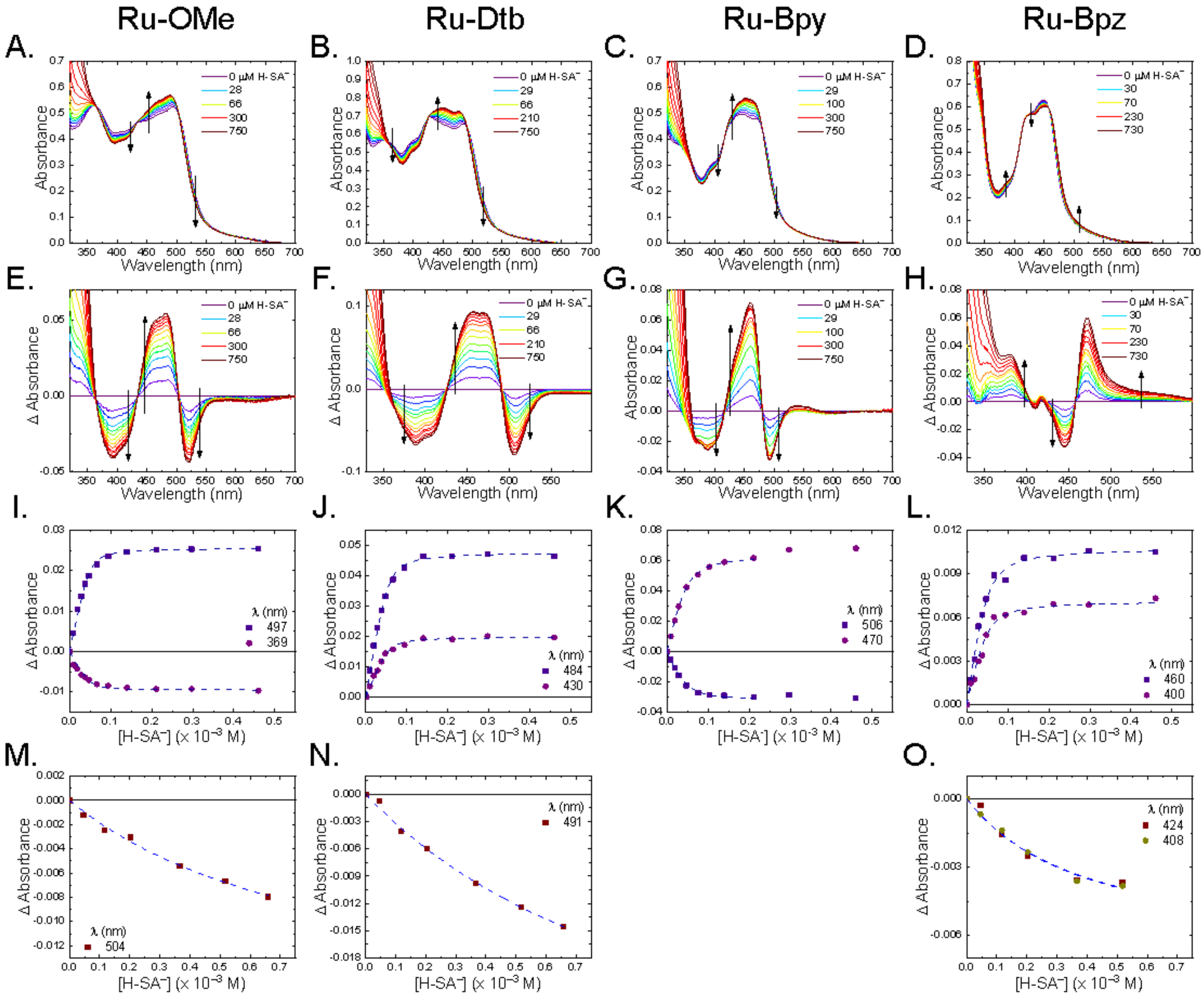

Figure S13. A.–D. UV-Vis absorption titrations of A. **Ru-OMe** (40 µM), B. **Ru-Dtb** (50 µM), C. **Ru-Bpy** (50 µM), and D. **Ru-Bpz** (50 µM) with the indicated H-SA⁻ concentrations in CH_3CN solutions. E.–H. Show the change in absorption for E. **Ru-OMe**, F. **Ru-Dtb**, G. **Ru-Bpy**, and H. **Ru-Bpz** in which the initial spectra with 0 µM H-SA⁻ was subtracted from the other spectra. Binding isotherms for the 1st ion pairing are shown, I. **Ru-OMe**, J. **Ru-Dtb**, K. **Ru-Bpy**, and L. **Ru-Bpz**; dashed lines are fits using a 1:1 binding isotherm.[6] A second ion pairing was modeled for both **Ru-OMe** (M.), **Ru-Dtb** (N.), and **Ru-Bpz** (O.).

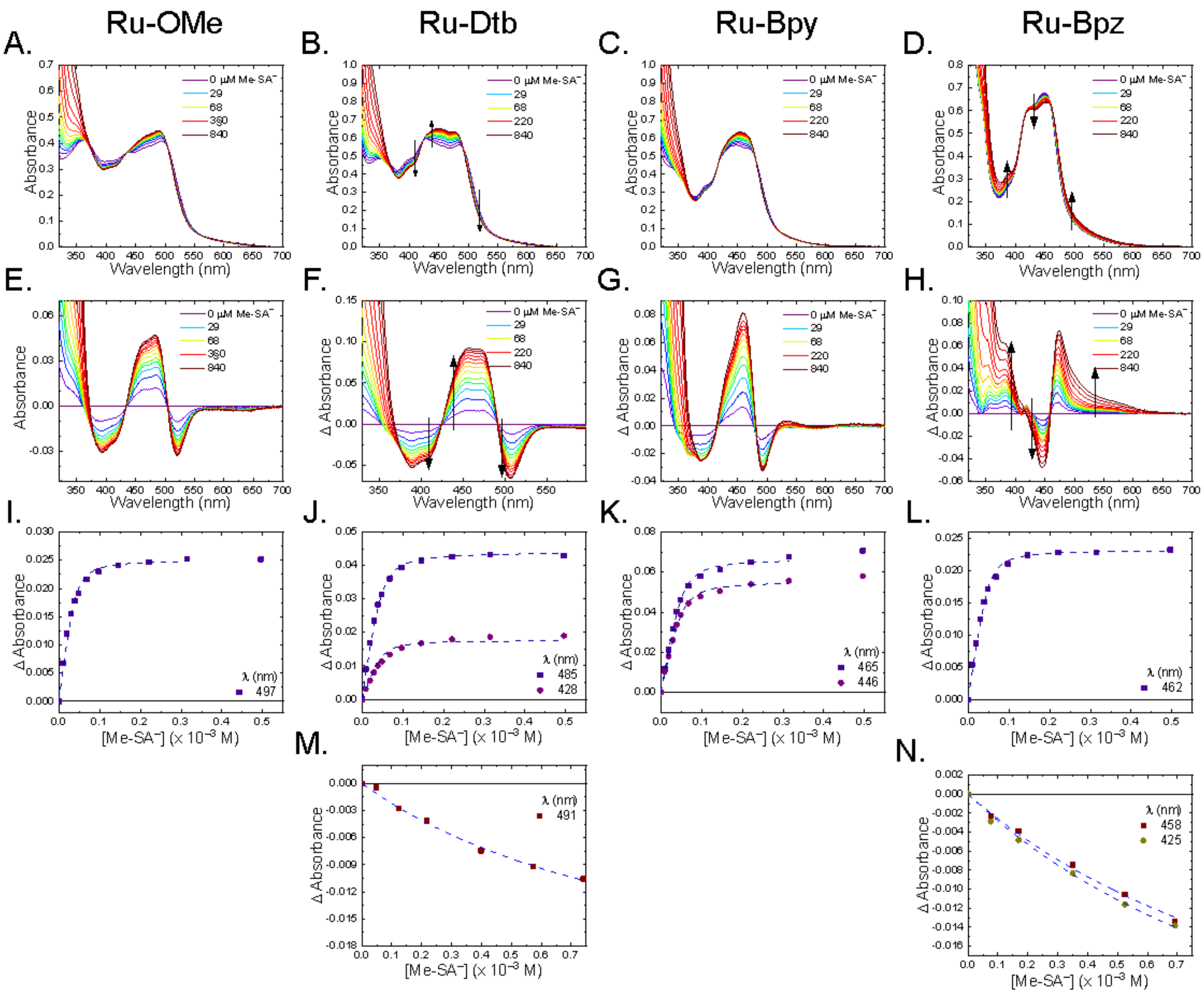

Figure S14. A.–D. UV-Vis absorption titrations of A. **Ru-OMe** (40 µM), B. **Ru-Dtb** (50 µM), C. **Ru-Bpy** (50 µM), and D. **Ru-Bpz** (50 µM) with the indicated Me-SA$^-$ concentrations in CH_3CN solutions. E.–H. Show the change in absorption for E. **Ru-OMe**, F. **Ru-Dtb**, G. **Ru-Bpy**, and H. **Ru-Bpz** in which the initial spectra with 0 µM Me-SA$^-$ was subtracted from the other spectra. Binding isotherms for the 1st ion pairing are shown, I. **Ru-OMe**, J. **Ru-Dtb**, K. **Ru-Bpy**, and L. **Ru-Bpz**; dashed lines are fits using a 1:1 binding isotherm.[6] A second ion pairing was modeled for both **Ru-Dtb** (M.) and **Ru-Bpz** (N.).

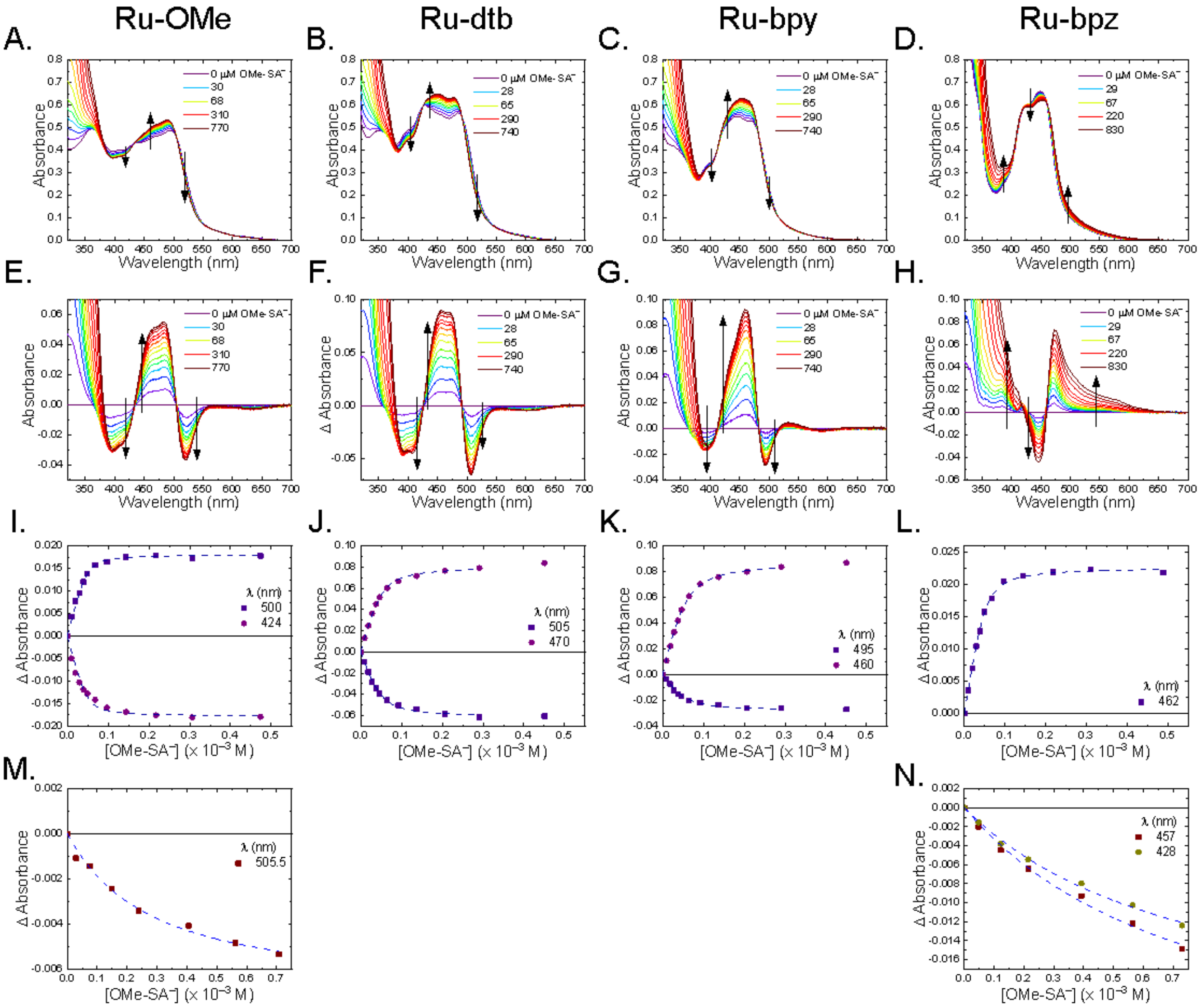

Figure S15. A.–D. UV-Vis absorption titrations of A. **Ru-OMe** (40 µM), B. **Ru-Dtb** (50 µM), C. **Ru-Bpy** (50 µM), and D. **Ru-Bpz** (50 µM) with the indicated OMe-SA$^-$ concentrations in CH_3CN solutions. E.–H. Show the change in absorption for E. **Ru-OMe**, F. **Ru-Dtb**, G. **Ru-Bpy**, and H. **Ru-Bpz** in which the initial spectra with 0 µM OMe-SA$^-$ was subtracted from the other spectra. Binding isotherms for the 1st ion pairing are shown, I. **Ru-OMe**, J. **Ru-Dtb**, K. **Ru-Bpy**, and L. **Ru-Bpz**; dashed lines are fits using a 1:1 binding isotherm.[6] A second ion pairing was modeled for both **Ru-OMe** (M.) and **Ru-Bpz** (N.).

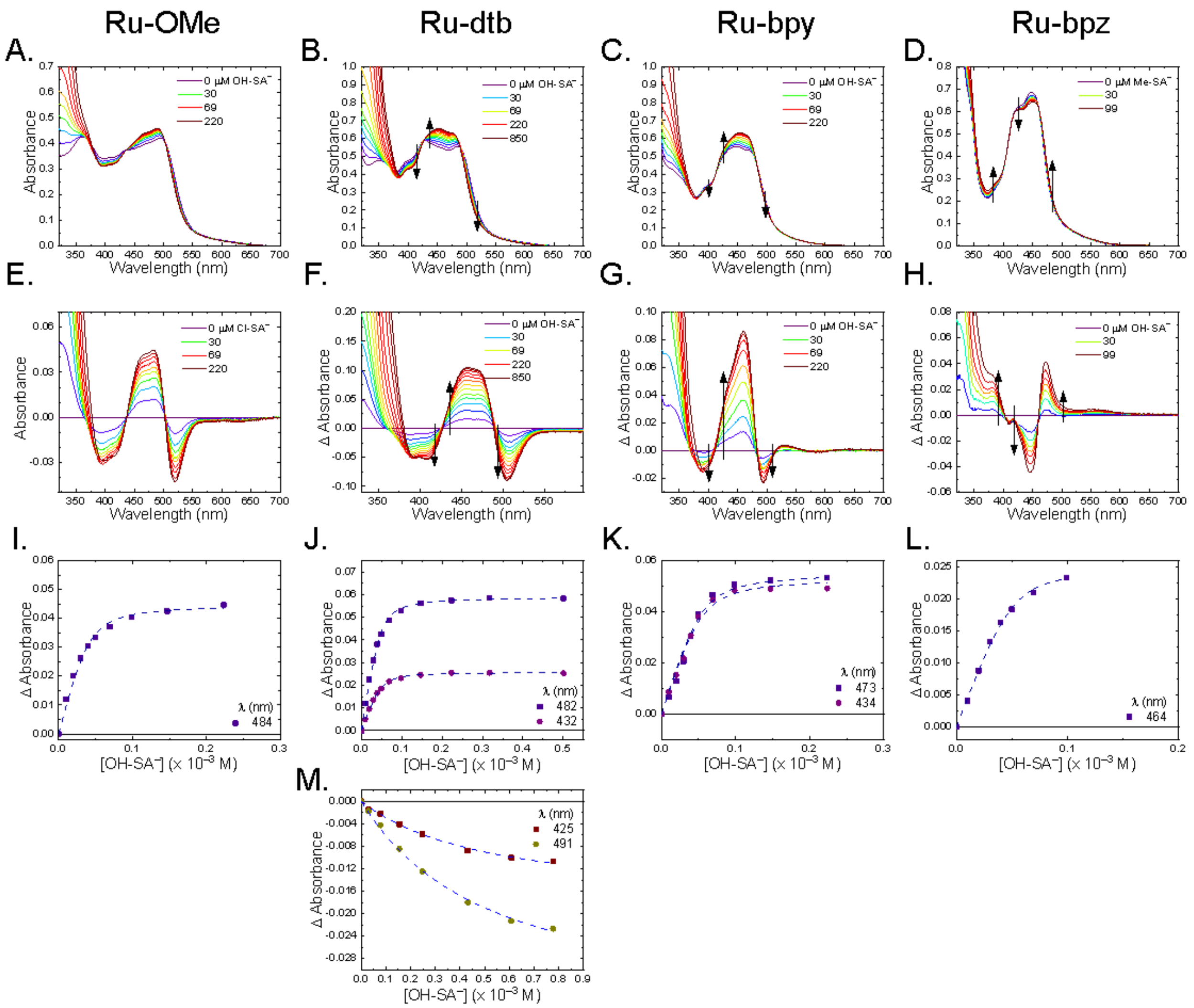

Figure S16. A.–D. UV-Vis absorption titrations of A. **Ru-OMe** (40 µM), B. **Ru-Dtb** (50 µM), C. **Ru-Bpy** (50 µM), and D. **Ru-Bpz** (50 µM) with the indicated OH-SA⁻ concentrations in CH_3CN solutions. E.–H. Show the change in absorption for E. **Ru-OMe**, F. **Ru-Dtb**, G. **Ru-Bpy**, and H. **Ru-Bpz** in which the initial spectra with 0 µM OH-SA⁻ was subtracted from the other spectra. Binding isotherms for the 1st ion pairing are shown, I. **Ru-OMe**, J. **Ru-Dtb**, K. **Ru-Bpy**, and L. **Ru-Bpz**; dashed lines are fits using a 1:1 binding isotherm.[6] A second ion pairing was modeled for both **Ru-Dtb** (M.).

Transient Spectroscopy:

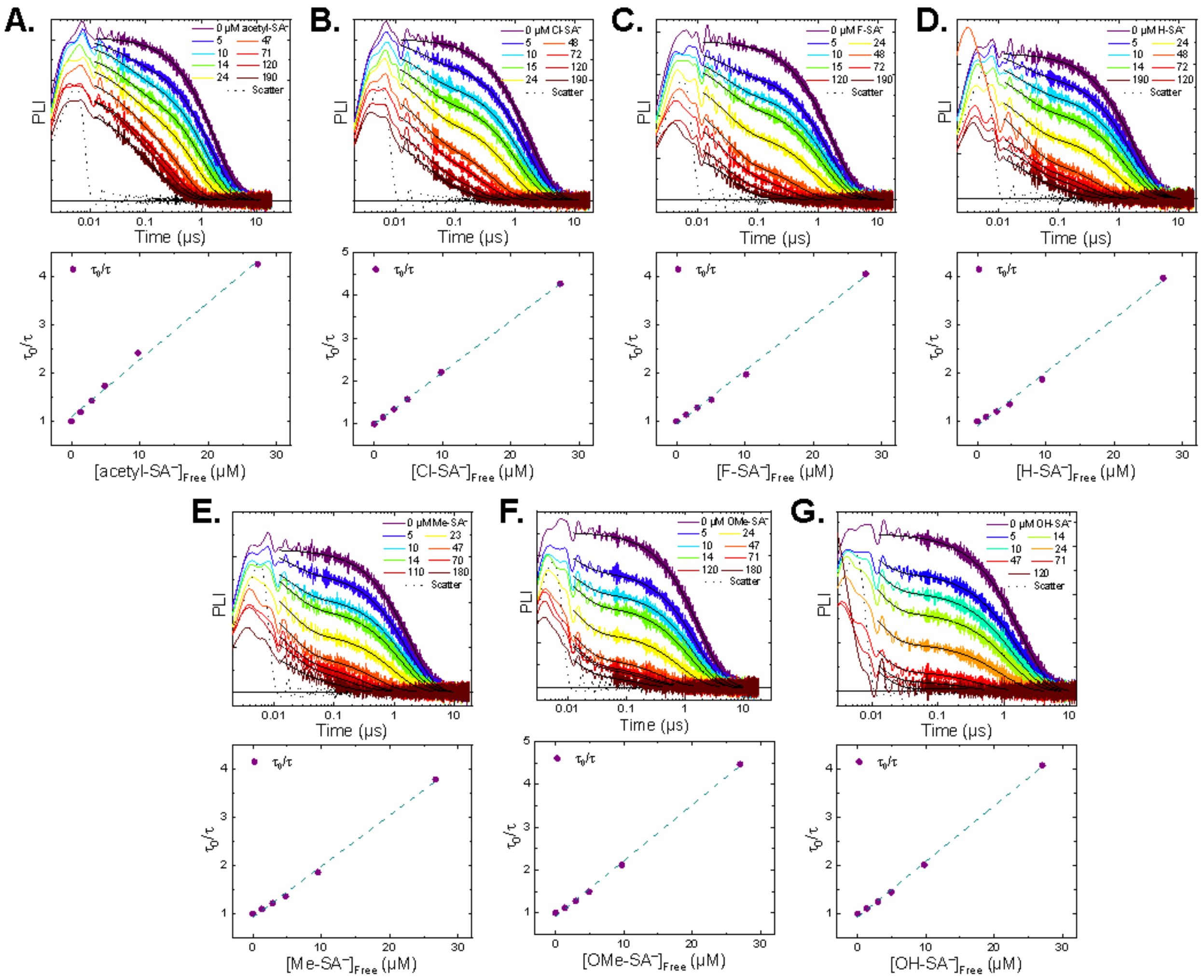

Figure S17. Time-resolved photoluminescence titration of **Ru-Bpz** (~25 μM) with the indicated concentrations of R-SA⁻ derivatives (Top) and Stern–Volmer plots for the diffusional quenching pathway (bottom), corrected for the bound concentration of R-SA⁻. A) **acetyl-SA⁻**, B) **Cl-SA⁻**, C) **F-SA⁻**, D) **H-SA⁻**, E) **Me-SA⁻**, F) **OMe-SA⁻**, G) **OH-SA⁻**. Dotted black trace is the instrument response function, recorded by scattering the laser into the detection system with no sample present. Changes in the initial intensities (times <10 ns) are due mainly to changes in the ground state absorbance at 460 nm from ion-pairing.

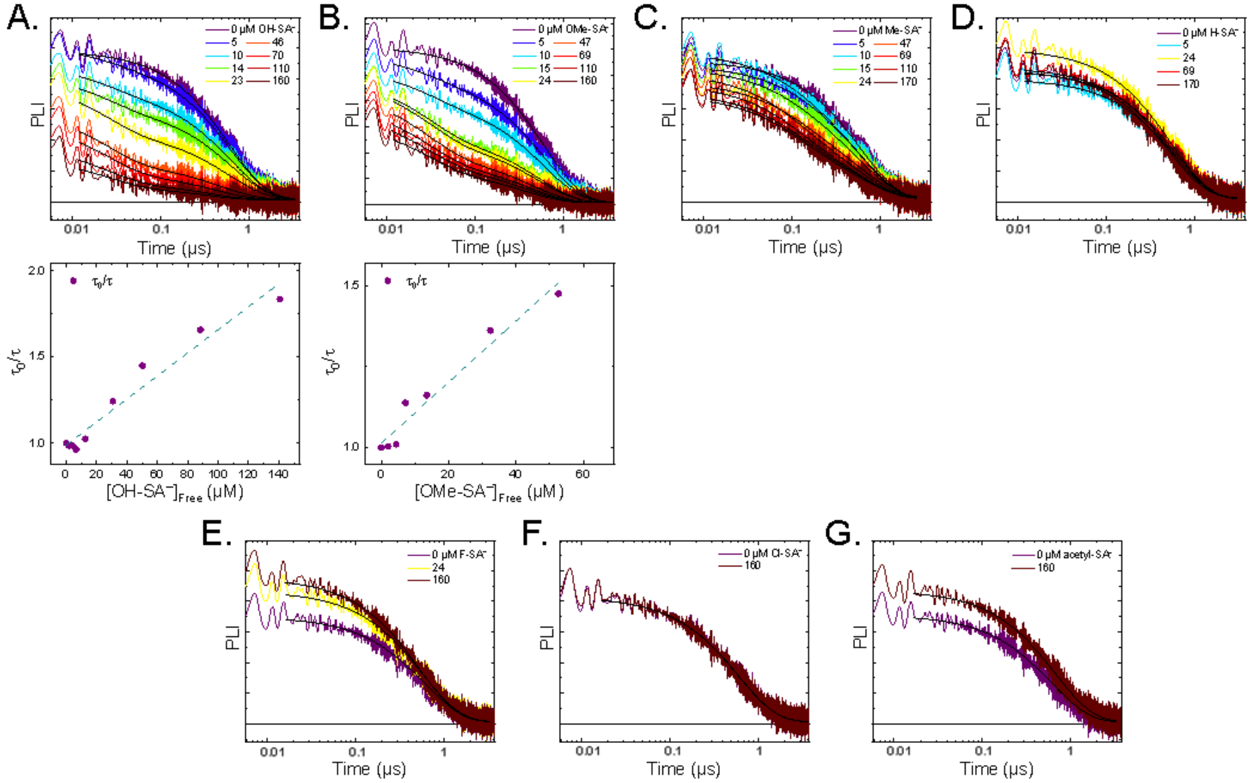

Figure S18. Time-resolved photoluminescence titration of **Ru-Bpy** (~25 μM) with the indicated concentrations of R-SA⁻ derivatives (Top) and Stern–Volmer plots for the diffusional quenching pathway (bottom), corrected for the bound concentration of R-SA⁻. A) **OH-SA⁻**, B) **OMe-SA⁻**, C) **Me-SA⁻**, D) **H-SA⁻**, E) **F-SA⁻**, F) **Cl-SA⁻**, G) **acteyl-SA⁻**. Changes in the initial intensities (times <10 ns) for **OH-**, **OMe-SA⁻**, and **Me-SA⁻** are due to the increased rate of quenching by the ion-paired salicylate. For the other derivatives, the increase in absorbance comes mainly to changes in the ground state absorbance at 460 nm from ion-pairing.

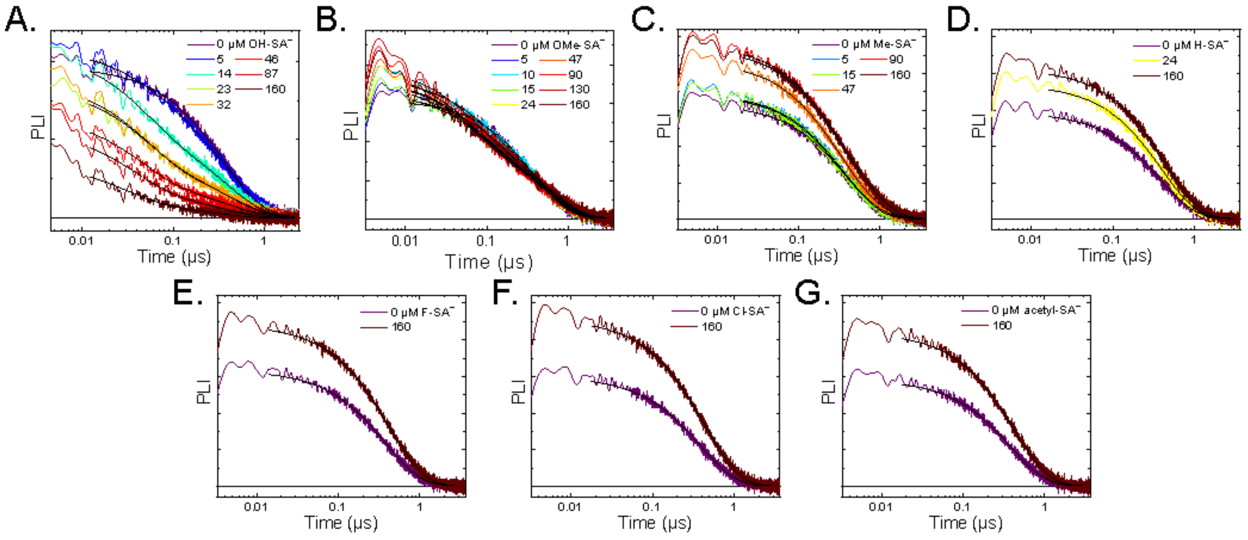

Figure S19. Time-resolved photoluminescence titration of **Ru-Dtb** (~25 μM) with the indicated concentrations of R-SA⁻ derivatives. A) **OH-SA⁻**, B) **OMe-SA⁻**, C) **Me-SA⁻**, D) **H-SA⁻**, E) **F-SA⁻**, F) **Cl-SA⁻**, G) **acteyl-SA⁻**. Changes in the initial intensities (times <10 ns) for **OH-** are due to the increased rate of quenching by the ion-paired salicylate. For the other derivatives, the increase in absorbance comes mainly to changes in the ground state absorbance at 460 nm from ion-pairing.

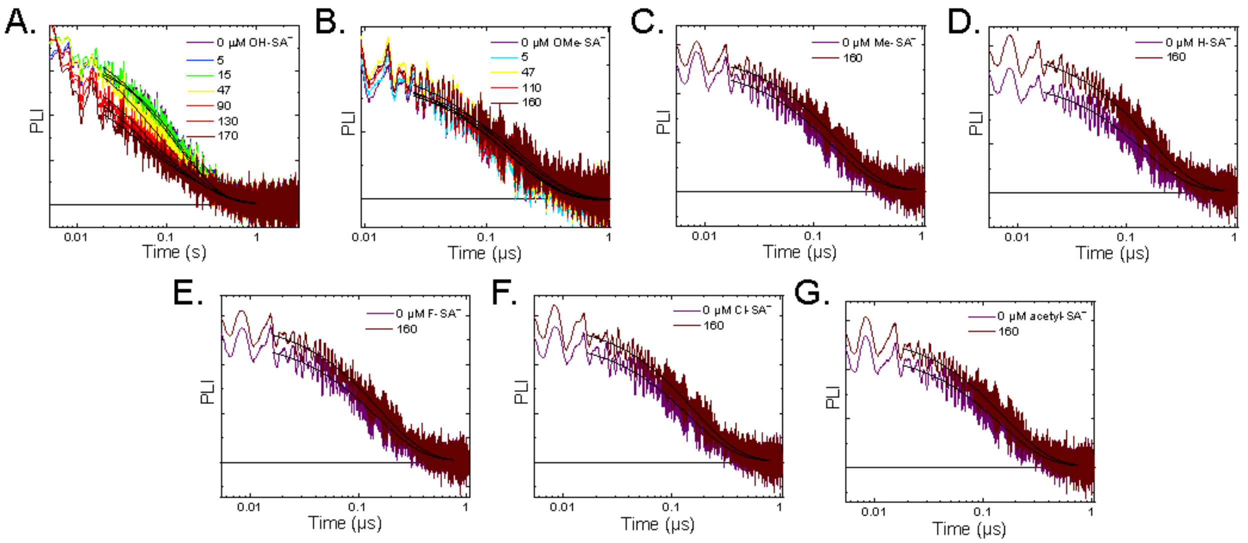

Figure S20. Time-resolved photoluminescence titration of **Ru-Dtb** (~25 μM) with the indicated concentrations of R-SA⁻ derivatives. A) **OH-SA⁻**, B) **OMe-SA⁻**, C) **Me-SA⁻**, D) **H-SA⁻**, E) **F-SA⁻**, F) **Cl-SA⁻**, G) **acteyl-SA⁻**.

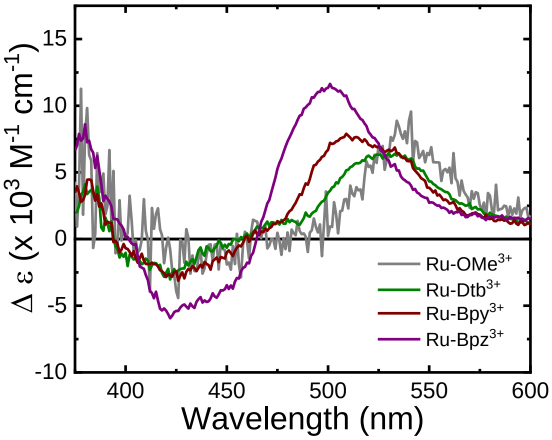

Figure S21. Reduced ruthenium (Ru^{3+}) spectra for the indicated **Ru-LL** complexes, determined through previously described methods.[7]

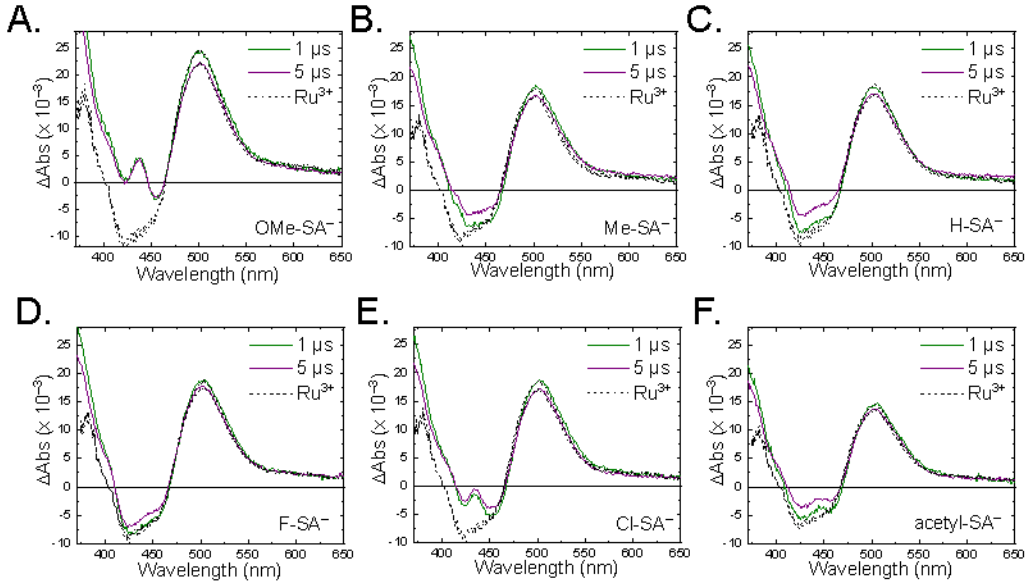

Figure S22. Transient absorption full spectra of **Ru-Bpz** (~25 μM) in the presence of the indicated R-SA⁻ (1 eq.) derivatives at the indicated time delays; A) **OMe-SA⁻**, B) **Me-SA⁻**, C) **H-SA⁻**, D) **F-SA⁻**, E) **Cl-SA⁻**, and F) **acteyl-SA⁻**. **OH-SA⁻** could not be recorded due to **Ru-Bpz** degradation on the timescale needed to collect the data. The reduced ruthenium complex normalized to the maximum of the reduced signal in the transient absorption around 510 nm is shown as the dotted black line.

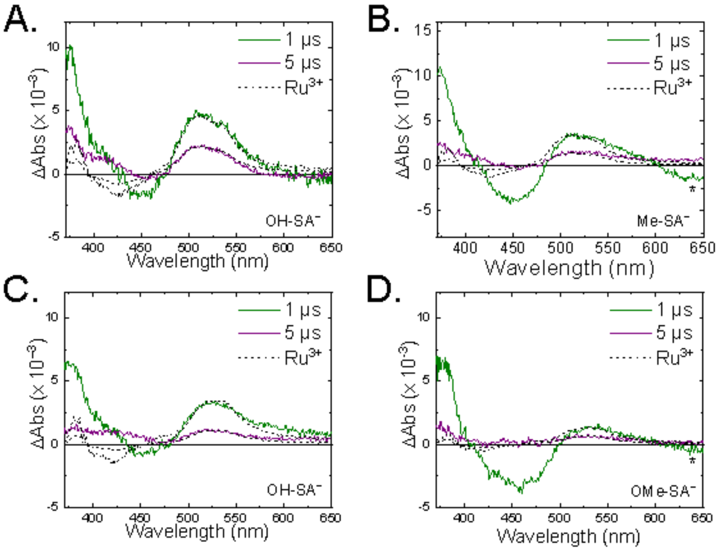

Figure S23. Transient absorption full spectra of **Ru-Bpy** (A and B) and **Ru-Dtb** (C and D), ~25 μM, in the presence of the indicated R-SA^{-} (1 eq.) at the indicated time delays. The reduced ruthenium complex normalized to the maximum of the reduced signal in the transient absorption around 510 nm is shown as the dotted black line. The asterisk denotes uncorrected emission. Due to this unquenched excited state a significant absorbance is measured at wavelengths below 400 nm, convoluting observation of the phenol radical. At 5 μs, the excited-state has decayed away and the remaining absorbance can be assigned to a mixture of the reduced ruthenium complex and phenol radical.

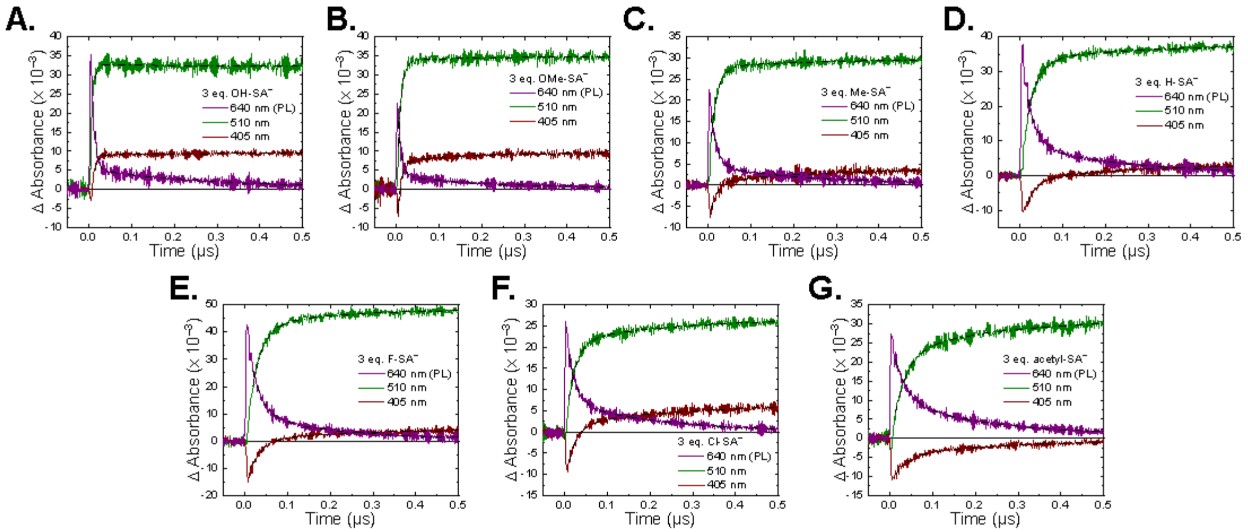

Figure S24. Transient absorption kinetics of **Ru-Bpz**, ~25 μM, in the presence of the indicated R-SA⁻ derivatives (3 eq.) recorded at 405 and 510 nm with overlaid fits to a biexponential equation; A) **OH-SA⁻** B) **OMe-SA⁻**, C) **Me-SA⁻**, D) **H-SA⁻**, E) **F-SA⁻**, F) **Cl-SA⁻**, and G) **acteyl-SA⁻**. 405 nm data corresponds to a mixture of the ground state recovery and growth of the phenol radical. Time-resolved photoluminescence, arbitrary units, recorded at 640 nm is shown in purple.

^{1}H NMR

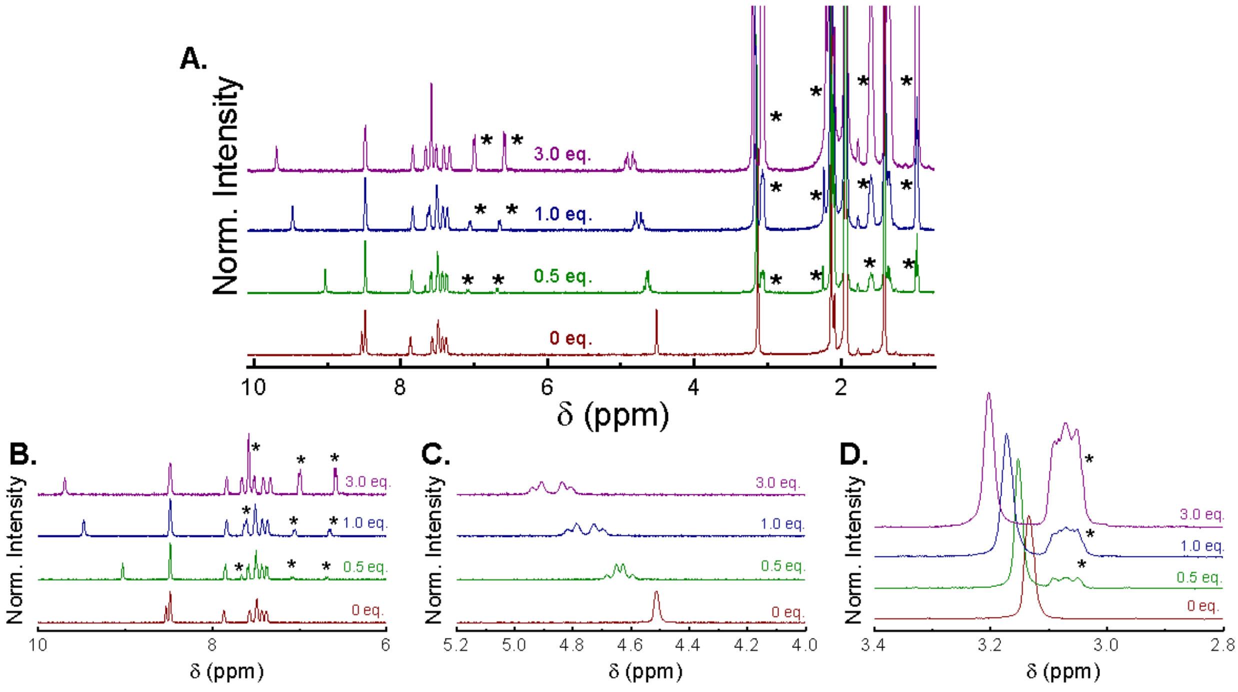

Figure S25. ^{1}H Nuclear magnetic resonance spectra recorded in CD_3CN for **Ru-Dtb** upon the addition of up to 3 eq. of **Me-SA$^-$**. A) The full spectra from 1–10 ppm. B) The aromatic region. C) The methylene (CH_2-) resonances on the tmam ligand. D) The methyl (CH_3-) resonances on the tmam ligand. Asterisks denote proton resonances from **Me-SA$^-$** and the tetrabutylammonium cation.

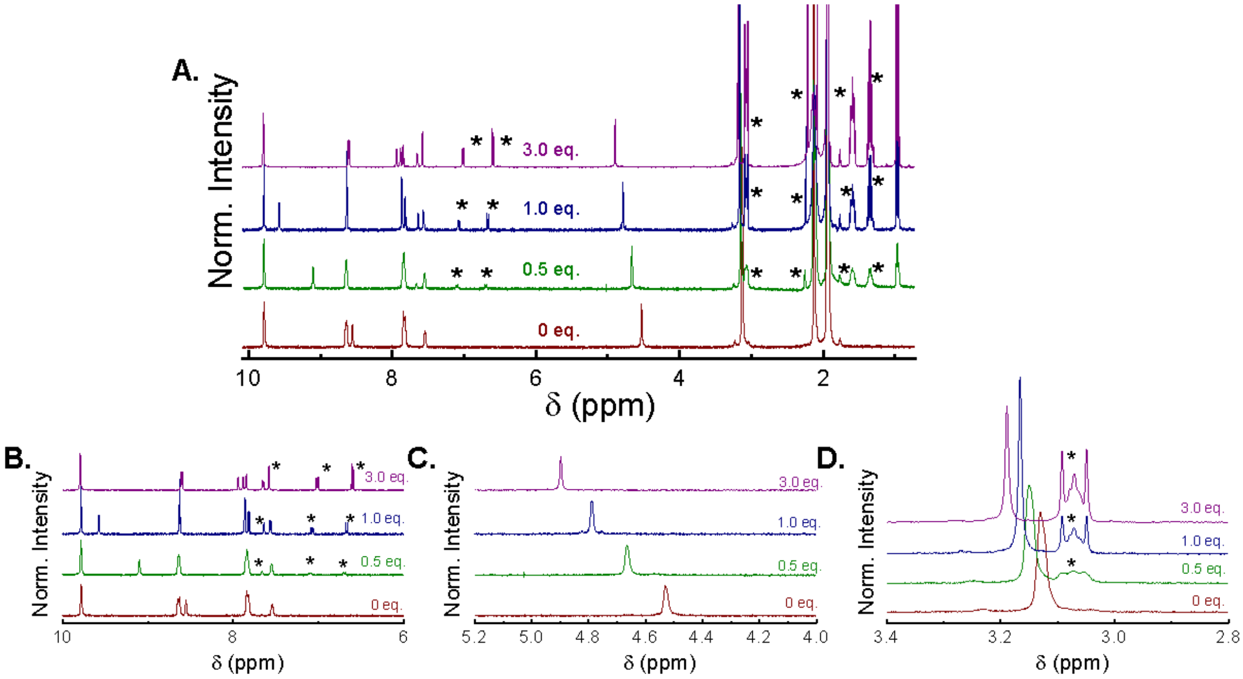

Figure S26. [1]H Nuclear magnetic resonance spectra recorded in CD_3CN for **Ru-Bpy** upon the addition of up to 3 eq. of **Me-SA⁻**. A) The full spectra from 1–10 ppm. B) The aromatic region. C) The methylene (CH_2-) resonances on the tmam ligand. D) The methyl (CH_3-) resonances on the tmam ligand. Asterisks denote proton resonances from **Me-SA⁻** and the tetrabutylammonium cation.
